# A Novel Mechanism of Optical Spectrum Formation in the Cs_2_NaInCl_6_ Doped with Sb and Er

**DOI:** 10.1021/acs.chemmater.5c03256

**Published:** 2026-05-28

**Authors:** Inna A. Ivashchenko, Małgorzata Makowska-Janusik, Lubomir D. Gulay, Yurij G. Kazarinov, Karina V. Lamonova, Yevheniia Smortsova, Anatoli I. Popov, Katarzyna Matras-Postołek

**Affiliations:** † Cracow University of Technology, Faculty of Chemical Engineering and Technology, Warszawska St. 24, Cracow 31-155, Poland; ‡ Faculty of Science and Technology, 69695 Jan Długosz University, Al. Armii Krajowej 13/15, Czestochowa 42-200, Poland; § Lesya Ukrainka Volyn National University, Voli Ave. 13, Lutsk 43000, Ukraine; ∥ National Science Center Kharkiv Institute of Physics and Technology, Akademichna St. 1, Kharkiv 61108, Ukraine; ⊥ Donetsk Institute for Physics and Engineering named after O.O. Galkin, NAS of Ukraine, Nauky Ave. 46, Kyiv 03028, Ukraine; # 9179 Deutsches Elektronen Synchrotron DESY, Notkestr. 85, Hamburg 22607, Germany; ∇ Institute of Solid State Physics, University of Latvia, Riga LV-1063, Latvia

## Abstract

We present a mechanism describing the formation of the optical spectrum in double halide perovskites (DHPs), specifically Sb- and Er-doped Cs_2_NaInCl_6_. Optical measurements conducted at the Deutsches Elektronen-Synchrotron (DESY) facility between 10 and 300 K have revealed that the bandgap of Cs_2_NaInCl_6_ is significantly larger than previously estimated, measuring 6.7 eV. Based on density-functional theory (DFT) calculations, we conclude that the formation of In^+^ (5s^2^ optical center) is crucial to the luminescence observed in Cs_2_NaInCl_6_ microcrystals produced by precipitation techniques. The formation of In^+^ is associated with the transfer of electron density from Cl^–^ to In^3+^, which occurs due to defects in Cs_2_NaInCl_6_, resulting in increased In content. This process is linked to the self-trapped exciton mechanism and the formation of defect-trapping levels within the Cs_2_NaInCl_6_ bandgap. Additionally, embedding Sb^3+^ (5s^2^ doped ion) and Er^3+^ affects both the crystal structure and absorption properties of Cs_2_NaInCl_6_ samples. These findings shed light on the intricate interactions between the synthesis, composition, and the optical behavior of both undoped and Sb^3+^, Er^3+^-doped Cs_2_NaInCl_6_ powder samples.

## Graphical abstract

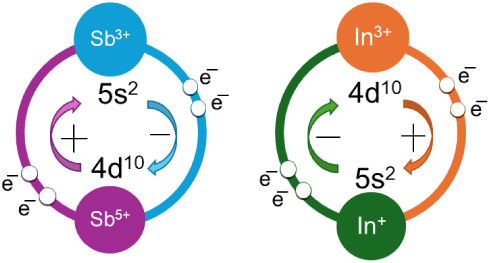

## Introduction

Halide double perovskites (HDPs), characterized by broadband emission, can have significant applications in various optoelectronic fields.^1,2^ Specifically, white perovskite light-emitting diodes (LEDs) obtained this way can be utilized for indoor illumination. The primary advantage of HDPs is their ability to produce white light in a single component.^3,4^ This strategy is more successful than the use of multiple phosphors in LED production.

HDPs with the general formula A^I^_2_M^I^M^III^X_6_ (where A^I^ = Rb^+^, Cs^+^; M^I^ = Na^+^, K^+^, Cu^+^, Ag^+^; M^III^ = In^3+^, Sb^3+^ or Bi^3+^; X = Cl^–^, Br^–^, or I^–^) feature soft lattices and strong exciton–phonon coupling. The compositional design of the HDPs induces Jahn–Teller distortions, promoting the formation of self-trapped excitons (STEs) and resulting in a broadband emission with high efficiency.^5^ STEs are electron–hole pairs that bind a carrier and they are associated with lattice deformation. The STEs lose some energy after self-trapping; therefore, their emission wavelengths are longer than their excitation wavelengths, caused by the bandgap of the HDPs. That is the reason for the large Stokes shift in STE emission.^6^

Cs_2_NaInCl_6_ (CNIC) is one of the most interesting candidates for investigating the optical properties of HDPs because of its direct bandgap, which varies among authors from 4.3 to 5.1 eV.^7–9^ Nevertheless, the optically forbidden transitions observed in CNIC and related to dark STEs reduce the intensity of the intrinsic photoluminescence (PL), thereby limiting its practical use in optoelectronics.^10^

Chemical doping is an effective strategy for breaking the parity-forbidden transitions in CNIC-based samples. Specifically, doping with ns^2^ ions, like Sb^3+^, creates suitable active sites and coordination environments that facilitate ^3^P_n_ (n = 0, 1, 2) → ^1^S_0_ transitions. Another way to modify CNIC excitation in the near-infrared (NIR) range is to dope it with rare-earth (RE) metals that prefer octahedral positions. Through multiple doping of Sb^3+^ and Er^3+^, it is possible to control the emission wavelength, intensity, and lifetime.^10–14^ These materials could be highly advantageous for solid-state lighting, gamma-ray detection, and other optical applications.^1,2^

The main drawback of such multiple doping is that the origin of broadband emission becomes unclear, specifically due to the presence of two different emission mechanisms associated with STE and/or ns^2^ ions.^15^ Although substantial efforts have been made to identify the structural origins and photophysical processes of electron excitation and relaxation, there is an increasing need for a systematic and comprehensive investigation of the emission mechanism in CNIC, including theoretical DFT calculations. The knowledge obtained from such studies is essential to the controllable design of the CNIC-based luminescence materials for various applications.

## Experimental Section

### Materials

CsCl (99.5%), NaCl (99.9%), InCl_3_ (99.99%), SbCl_3_ (99.95%), ErCl_3_ (99.9%), and 36% HCl solution were sourced from Sigma-Aldrich (USA) and used as received.

### Preparation of the Precursors and Synthesis

Solution A was made by dissolving 4 or 4.5 mmol of InCl_3_ in 10 mL of 36% HCl. Solution B was made by dissolving 1 mmol SbCl_3_ in 10 mL of 36% HCl. Solution C was made by dissolving 10 mmol CsCl in 5 mL of 36% HCl. The calculated mass of NaCl or ErCl_3_ (Table 1) was then added into 50 mL glass containers with 10 mL of 36% HCl. The obtained mixtures were heated to 70 °C. When all salts dissolved, solution A or B (depending on the composition) was added. The mixtures were then stirred for 30 min, after which 1 mL of solution C was added to each container. White precipitates formed immediately (crystal size <100 μm). Next, the containers were cooled in air while stirring the powders for 2 h (or 20 min for Cs_2_NaInCl_6_). The precipitates were filtered and washed with anhydrous ethanol to remove the acid. They were then calcined for 2 h at 160 °C to remove any remaining acid and water (Table 1, Figures 1 and S1). Using this precipitation method, Cs_2_NaInCl_6_ (CNIC) and doped samples (Cs_2_NaIn_0.9_Sb_0.1_Cl_6_ and Cs_2_NaIn_0.6_Sb_0.1_Er_0.3_Cl_6_) were synthesized as microcrystalline powders, based on the following reaction:

$$\mathrm{NaCl}+{\mathrm{InCl}}_{3}+\left({\mathrm{SbCl}}_{3}\right)+\left({\mathrm{ErCl}}_{3}\right)\frac{T,K}{\mathrm{dissolut.}}+\mathrm{CsCl}\to {\mathrm{Cs}}_{2}{\mathrm{NaInCl}}_{6}:\mathrm{Sb}/\mathrm{Er}↓$$

**Figure 1:**
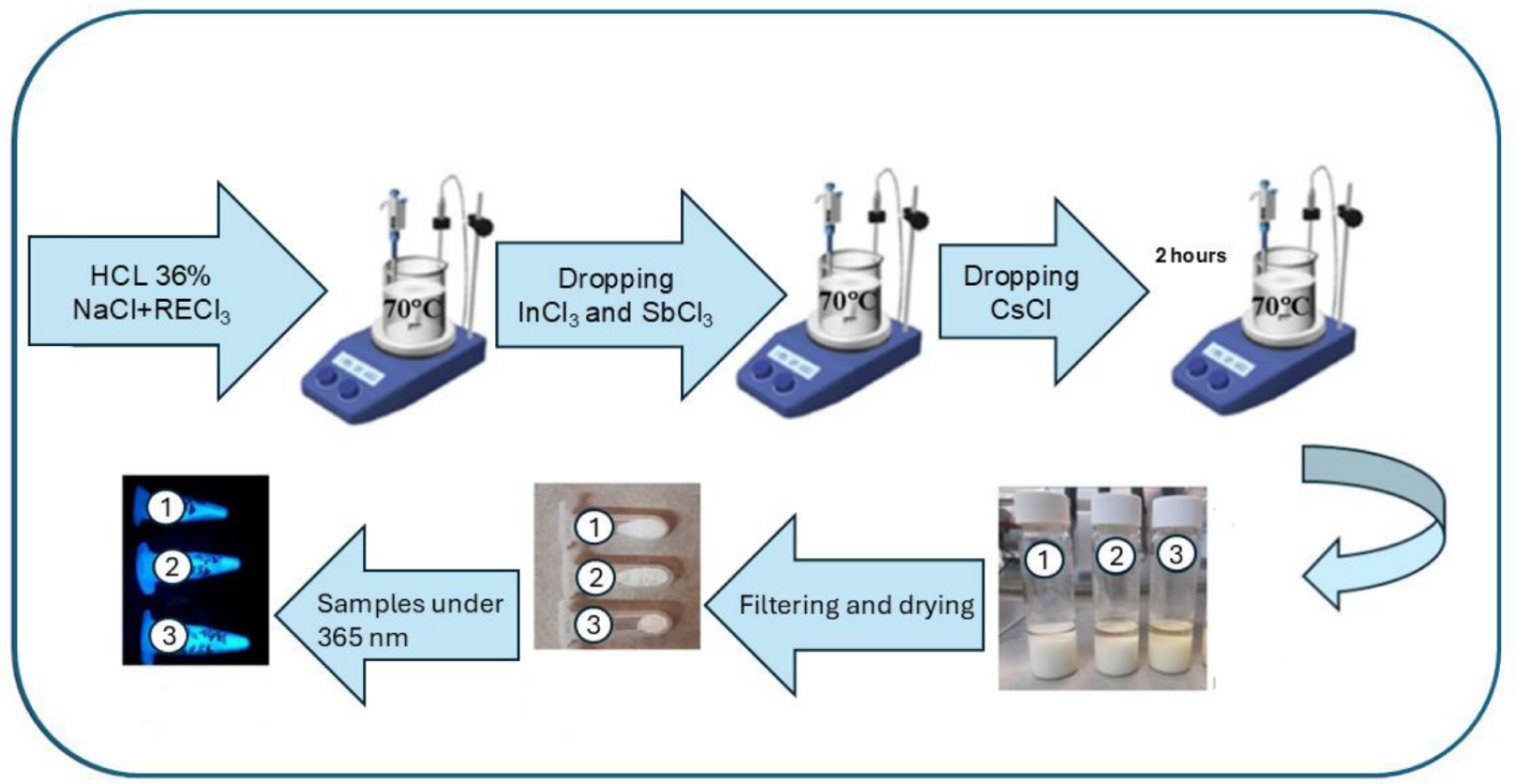
Schematic of the precipitation synthesis technique and final Sb/Er codoped Cs_2_NaInCl_6_ (CNIC) samples: 1—CNIC, 2—Cs_2_NaIn_0.9_Sb_0.1_Cl_6_, and 3—Cs_2_NaIn_0.6_Sb_0.1_Er_0.3_Cl_6_.

**Table 1:** Amount of the Chlorides Used for the Sample Synthesis and Yield of the Reaction Product

Composition	n, CsCl mmol	n, NaCl mmol	n, InCl_3_ mmol	n, SbCl_3_ mmol	n, ErCl_3_ mmol	m_pr_,^a^ g	η,^b^ %
Cs_2_NaInCl_6_; CNIC	2	1	1	-	-	0.4075	66
Cs_2_NaIn_0.9_Sb_0.1_Cl_6_	2	1	0.9	0.1	-	0.5204	84
Cs_2_NaIn_0.6_Sb_0.1_Er_0.3_Cl_6_	2	1	0.6	0.1	0.3	0.3870	61

a m_pr_—mass of the obtained samples.

b η—yield of the reaction product.

### Characterization Methods

X-ray photoelectron spectroscopy (XPS) was performed with a PHI VersaProbe II Scanning XPS system. Al Kα X-rays (1486.6 eV) were focused on a 100 μm spot and scanned over a 400 μm^2^ area. The spectra were analyzed with PHI MultiPak software (v.9.9.3) and the Shirley method was used to subtract the background. The XPS analysis has an information depth of about 5 nm in this setup. The element distribution in the samples was checked with Energy Dispersive X-ray Spectroscopy (EDS) using an Apreo 2S LoVac SEM (Thermo Fisher Scientific). The obtained samples were investigated with an X-ray powder diffraction (XRD) method using the SmartLab powder diffractometer (CuKα radiation, 2θ range of 10–120°, 0.05° scan step). The analysis of the XRD patterns was performed with a WinCSD software package (see Supporting Information). The absorption spectra were recorded using a Shimadzu UV-2600 UV–vis spectrophotometer. A comprehensive study of the PL properties within the UV–vis-NIR range was conducted using the P66 time-resolved VUV spectroscopy beamline at the PETRA III storage ring of DESY (Hamburg, Germany), covering 304 to 10.7 K. Additional fluorescence data at ambient temperature were collected using a Hitachi F-4600 spectrometer (see Supporting Information). Quantum chemical calculations were conducted within the framework of density functional theory (DFT) as implemented in the Vienna Ab Initio Simulation Package (VASP) (see Supporting Information). Additional detailed information on sample investigation methods is provided in the Supporting Information (SI); the main text focuses on the results obtained using these methods.

## Results and Discussion

The XPS analysis was conducted to determine the elemental composition, chemical bonding, and oxidation states of the chemical elements (see Figure 2). For all the samples, the Na 1s spectra were fitted with a single line centered at 1071.8 eV, indicating a Na^+^ oxidation state, which was consistent with previously reported data.^16^ For Cs, the samples exhibited 3d_5/2_ spectra centered at 724.2 eV, corresponding to the Cs^+^ oxidation state, aligning with.^17^ The 4d spectra in the Er-containing sample were analyzed using a doublet structure and the separation between the d_5/2_ and d_3/2_ doublets was 2.05 eV. The main 4d_5/2_ peak indicating the Er^3+^ oxidation state, was centered at 168.9 eV.^18^

**Figure 2:**
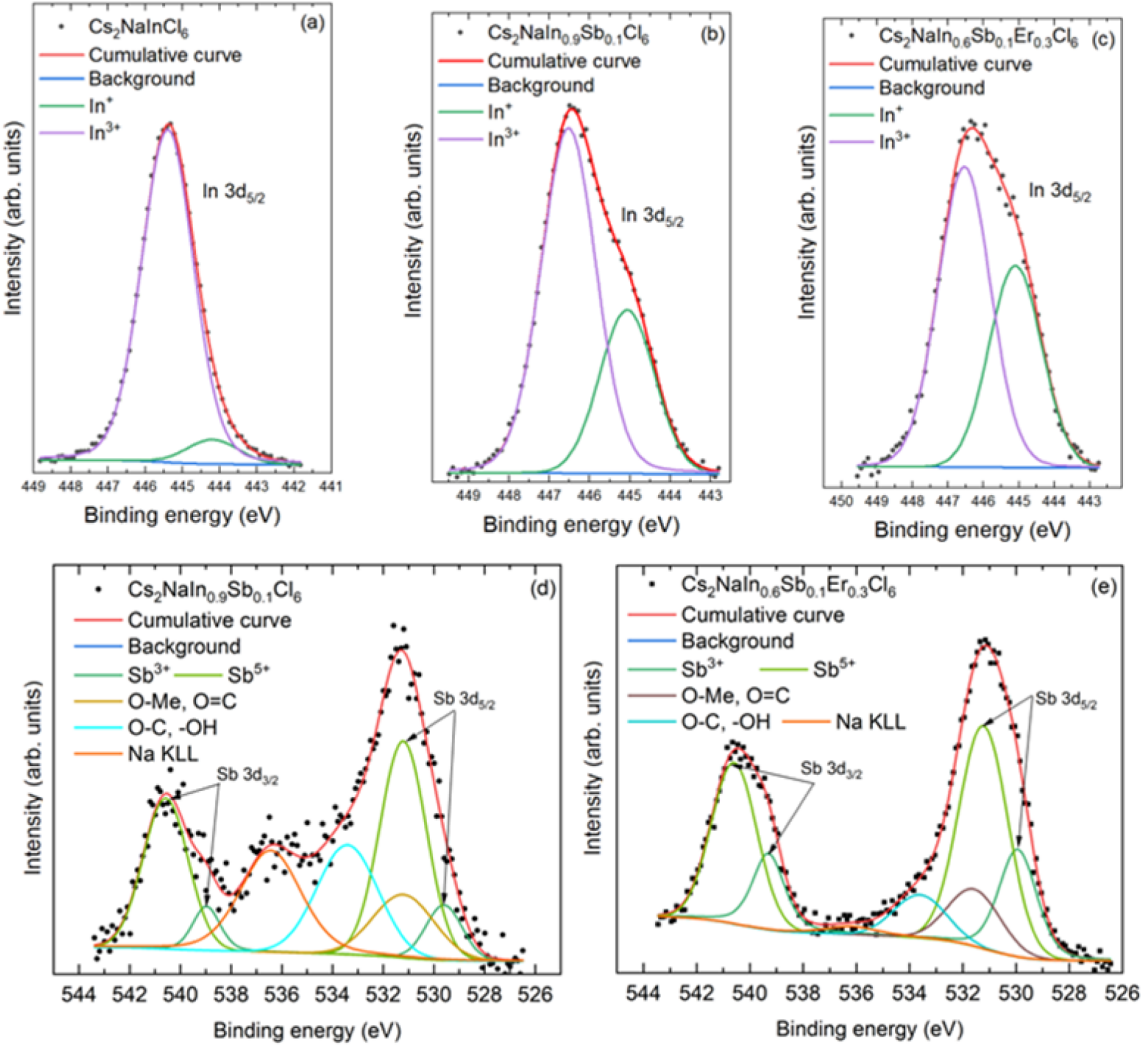
Chosen XPS spectra demonstrate the features of the Cs_2_NaInCl_6_ (a), Cs_2_NaIn_0.9_Sb_0.1_Cl_6_ (b, d), and Cs_2_NaIn_0.6_Er_0.3_Sb_0.1_Cl_6_ (c, e) samples.

The 3d_5/2_ spectra of the In ions from the Cs_2_NaInCl_6_, Cs_2_NaIn_0.9_Sb_0.1_Cl_6_, and Cs_2_NaIn_0.6_Er_0.3_Sb_0.1_Cl_6_ samples consisted of two lines centered at 445.07 and 446.5 eV (Figure 2 a,b,c). Based on ref 17 the presence of two In charge states, In^+^ and In^3+^, was identified. The intensities of the lines differed greatly, with In^3+^ being dominant. It should be mentioned that conducting a composition analysis in the case of the 3d_3/2_ line is not recommended because of the risk of significant error due to the wide bandgap of the studied samples. This analysis makes the background of inelastic losses much weaker within the first line 3d_5/2_ of the doublet than within the second 3d_3/2_. For the Sb-doped samples (Cs_2_NaIn_0.9_Sb_0.1_Cl_6_ and Cs_2_NaIn_0.6_Er_0.3_Sb_0.1_Cl_6_), the 3d spectra were analyzed and fitted with two doublet structures. The separation between the 3d_5/2_ and 3d_3/2_ was 9.34 eV. The first line of the 3d_5/2_ doublet was centered at 529.4 eV, indicating the presence of the Sb^3+^ charge state. The second line of the 3d_5/2_ doublet was positioned at 530.9 eV, which has been attributed to the Sb^5+^ charge state and it is consistent with ref 17 (Figure 2d). Additionally, some amounts of C and O have been detected due to the sample cleaning method with ethanol.

The XPS analysis gave intriguing results: the unexpected In^+^ and Sb^5+^ charge states were formed in the synthesized CNIC-based samples, which could significantly impact their optical properties, while the other elements existed in the predicted and stable Cs^+^, Na^+^, and Er^3+^ states.

The EDS elemental analysis is in line with the previously presented XPS results (Table 2) and indicates a deficiency in Na (9.3 at. % for CNIC) and higher concentrations of In (11 at. % for CNIC). This was caused by the presence of two valence states of In^+/3+^, which increased the amount of In in the samples instead of Na^+^, leading to its deficiency. The EDS results of Er for Cs_2_NaIn_0.6_Er_0.3_Sb_0.1_Cl_6_ (see the Supporting Information, Figures S1 and S2) showed a lower concentration (0.1 at. %), whereas the XPS result for the Er^3+^ concentration (2.7 at. %) aligned with the batch composition (3.0 at. %). Additionally, the concentrations of Cl were lower than the required stoichiometric amount for all samples. This discrepancy can be attributed to the distinct methodologies employed by the XPS and EDS techniques. For example, XPS is more suitable for identifying elements and their oxidation/bonding states in the top few nanometers (on the surface of the microcrystals). EDS allows checking the bulk composition: identifying which elements are present and their approximate concentrations within micrometer-scale regions. There could be a deviation in the concentration of elements when moving from a surface into the bulk.

**Table 2:** Analytical and Batch Compositions of the Synthesized Samples

Sample	Results	Cs^+^ (at. %)	Na^+^ (at. %)	In^+^/In^3+^ (at. %)	Er^3+^ (at. %)	Sb^3+^/Sb^5+^ (at. %)	Cl^–^ (at. %)
Cs_2_NaInCl_6_ (CNIC)	XPS	23.4	9.7	0.3/10.1	-	-	56.5
	EDS	21.0	9.3	10.5 (In^3+^)	-	-	59.2
	Batch comp.	20.0	10.0	10.0 (In^3+^)	-	-	60.0
Cs_2_NaIn_0.9_Sb_0.1_Cl_6_	XPS	21.9	9.7	0.6/8.8	-	0.4/0.6	58.0
	EDS	21.5	9.2	9.1 (In^3+^)	-	1.5 (Sb^3+^)	58.7
	Batch comp.	20.0	10.0	9.0 (In^3+^)	-	1.0 (Sb^3+^)	60.0
Cs_2_NaIn_0.6_Sb_0.1_Er_0.3_Cl_6_	XPS	22.5	7.6	2.0/4.1	2.7	0.4/0.9	59.8
	EDS	23.3	7.8	9.4 (In^3+^)	0.1	1.2 (Sb^3+^)	58.2
	Batch comp.	20.0	10.0	6.0 (In^3+^)	3.0	1.0 (Sb^3+^)	60.0

It was revealed that the undoped Cs_2_NaInCl_6_ and the Sb-doped sample crystallized with cubic symmetry and the Sp.Gr. *Fm*3̅*m* (Figure 3 and Table 3). Sb doping did not alter the cubic crystal structure, but it increased the unit cell size as compared with the undoped Cs_2_NaInCl_6_ sample.

**Figure 3:**
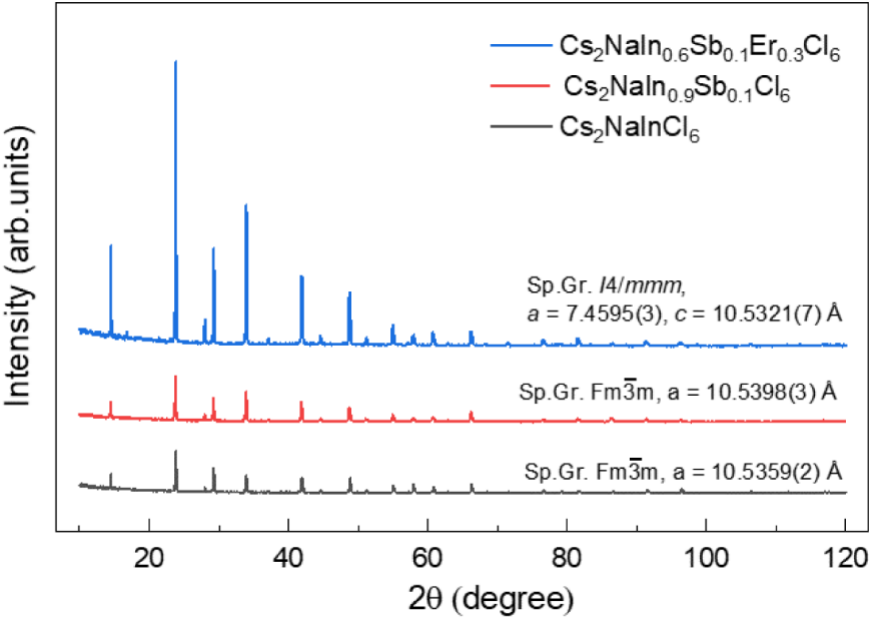
X-ray diffractograms of the undoped CNIC, Sb-doped, and codoped with Sb and Er CNIC samples.

**Table 3:** Compositions, Space Group, and Lattice Parameters of the Synthesized Powder Samples

Composition	Sp.Gr., lattice parameters (Å)
Cs_2_NaInCl_6_ (CNIC)	*Fm*3̅*m*, *a* = 10.5359(2)
Cs_2_NaIn_0.9_Sb_0.1_Cl_6_	*Fm*3̅*m*, *a* = 10.5398(3)
Cs_2_NaIn_0.6_Er_0.3_Sb_0.1_Cl_6_	*I*4/*mmm*, *a* = 7.4595(3), *c* = 10.5321(7)

Doping with both Sb and Er increased the lattice parameter and resulted in a structural transition from cubic to tetragonal symmetry characterized by the *I*4/*mmm* space group. The unit cell parameters agreed well with the ionic radii of the elements: In^3+^ (0.80 Å), Er^3+^ (0.89 Å), and Sb^3+^ (0.86 Å)^19,20^ and were consistent with previously reported data.^14^

The atomic coordinates of all tested samples were refined using the Rietveld method and WinCSD software,^21^ taking into account the previously discussed XPS and EDS results (Tables S1–S3). Two model structures with different compositions were proposed: stoichiometric Cs_2_NaInCl_6_ with In^3+^ and Cs_2_Na_0.97_(In^+^)_0.03_In^3+^Cl_6_ (Figure 4). We applied the same approach to Cs_2_NaIn_0.9_Sb_0.1_Cl_6_ with In^3+^ and Sb^3+^, and to Cs_2_Na_0.97_(In^+^)_0.03_(In^3+^)_0.9_(Sb^3+/5+^)_0.05_□_0.05_Cl_6_ (Figure 4), considering the different valence states of In^+/3+^ and Sb^3+/5+^ based on the XPS results. Because In^+^ and In^3+^ were distributed over different Wyckoff positions (Table S2), we used a formula with separate indices for In^+^ and In^3+^: ...(In^+^)_0.03_(In^3+^)_0.9_.... While Sb^3+/5+^ occupy the same 4a Wyckoff position, they had only one index in the formula with vacancies in the crystal structure for the charge balance: ...(Sb^3+/5+^)_0.05_□_0.05_.... Vacancies occurred in locations where M^III^ ions were typically found, aligning with the calculated interatomic distances of the samples (Table S3). As a result, the unit cell parameter increased, which was consistent with the ionic nature of the bonds in these samples. The presence of the V_M_^III^ vacancies diminished the electrostatic forces between the ions, resulting in a looser structure for Cs_2_Na_0.97_(In^+^)_0.03_(In^3+^)_0.9_(Sb^3+/5+^)_0.05_□_0.05_Cl_6_. Notably, the R_I_ and R_P_ parameters remained practically unchanged (Table S1). These results support our hypothesis that In^+^ cations can exist in the crystal structure of Cs_2_NaInCl_6_ and that Sb^5+^ is present in Sb-doped Cs_2_NaInCl_6_.

**Figure 4:**
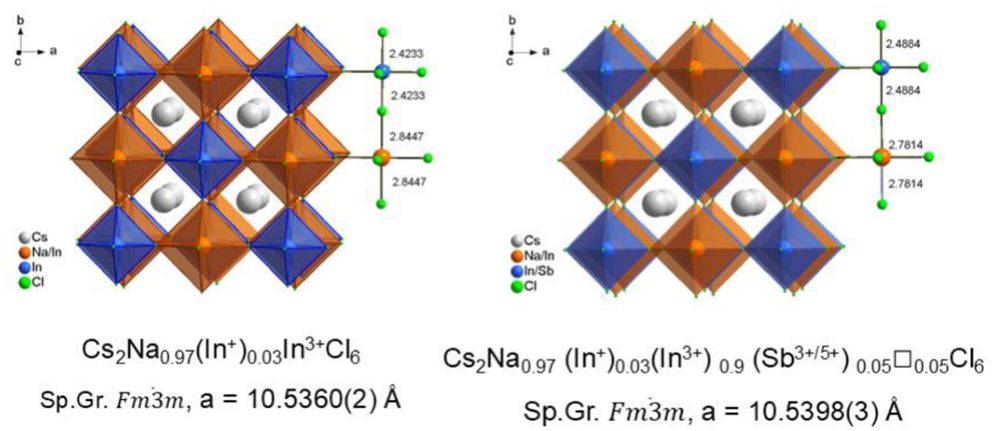
Models Cs_2_Na_0.97_(In^+^)_0.03_In^3+^Cl_6_ and Cs_2_Na_0.97_(In^+^)_0.03_(In^3+^)_0.9_(Sb^3+/5+^)_0.05_□_0.05_Cl_6_ for structures Cs_2_NaInCl_6_ and Cs_2_NaIn_0.9_Sb_0.1_Cl_6_ calculated using the Rietveld method.

To avoid random influence on the spectra shape, we measured spectra for samples prepared from other NaCl (e.g., ≥99.0%; from Merck (USA)) and with different annealing times (2–5 h). In all cases, we obtained Cs_2_NaInCl_6_ spectra which were very similar to those presented in Figure 5. An intense band observed at 230 nm (5.39 eV) in the absorption spectra of the CNIC sample is typically considered the absorption edge (Figure 5) and is consistent with previous reports.^7–9^ To avoid the unexpected influence of the reagents, the duration of the synthesis, and the time of powder annealing, etc., on the measured spectra, we performed the synthesis of the Cs_2_NaInCl_6_ several times (see Supporting Information). The high-energy optical measurements performed at DESY indicated that the absorption edge occurred at about 186 nm, implying a wider bandgap of 6.7 eV for the CNIC sample (see DFT Calculation Results section). Additionally, weaker absorption peaks were observed at 315, 360, 405, and 465 nm. Doping with Sb shifted the absorption spectrum to 250 nm, also altering the additional weaker bands (Figure 5). A similar complex band structure has previously been reported for the CNIC sample in report,^22^ although it remains unexplained.

**Figure 5:**
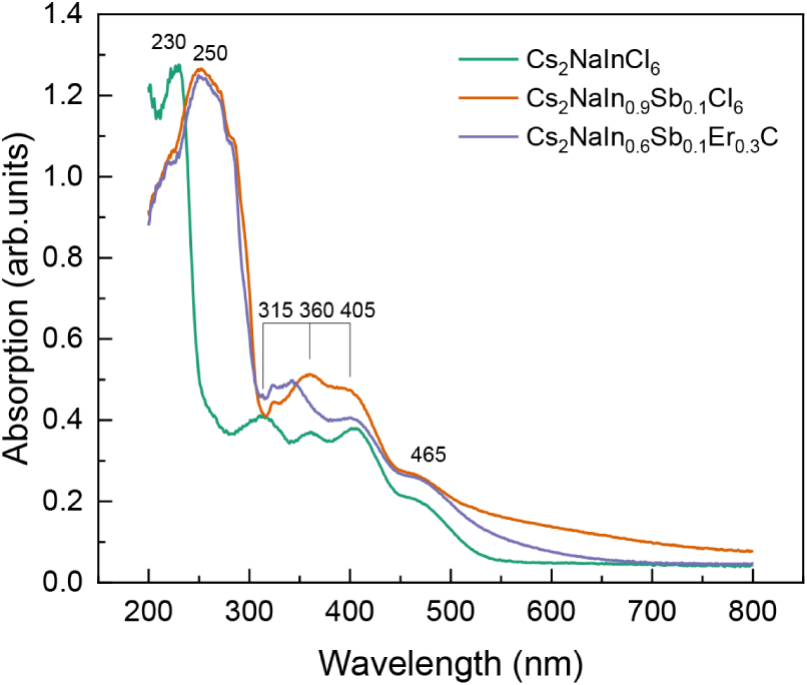
Absorption spectra measured for the undoped CNIC, Sb-doped (Cs_2_NaIn_0.9_Sb_0.1_Cl_6_), and codoped with Sb and Er CNIC (Cs_2_NaIn_0.6_Er_0.3_Sb_0.1_Cl_6_) samples.

Figure 6 shows the two-dimensional (2D) PLE maps plotting the PL intensity in the 300–1000 nm range on a color scale as a function of the excitation wavelength of the CNIC ([Fig fig6]a and 6d), Sb-doped ([Fig fig6]b and 6e), and codoped with Sb and Er ([Fig fig6]c and 6f) samples at the 304 and 10.7 K temperatures. All the 2D PLE maps at 10.7 K exhibited broad “white” luminescence (500–1000 nm) when excited in the range of ∼160–225 nm. The luminescence observed at 10.7 K remained unchanged with the Sb or Er incorporation. In the Sb-contained samples, strong luminescence bands were present: one band appeared at 450 nm with an excitation maximum between 270 and 280 nm, and another at 560 nm with an excitation maximum of approximately 300 nm. While the addition of Er introduced luminescence characteristics of f–f transitions within the RE ion, it did not lead to any other significant changes in the spectra.

**Figure 6:**
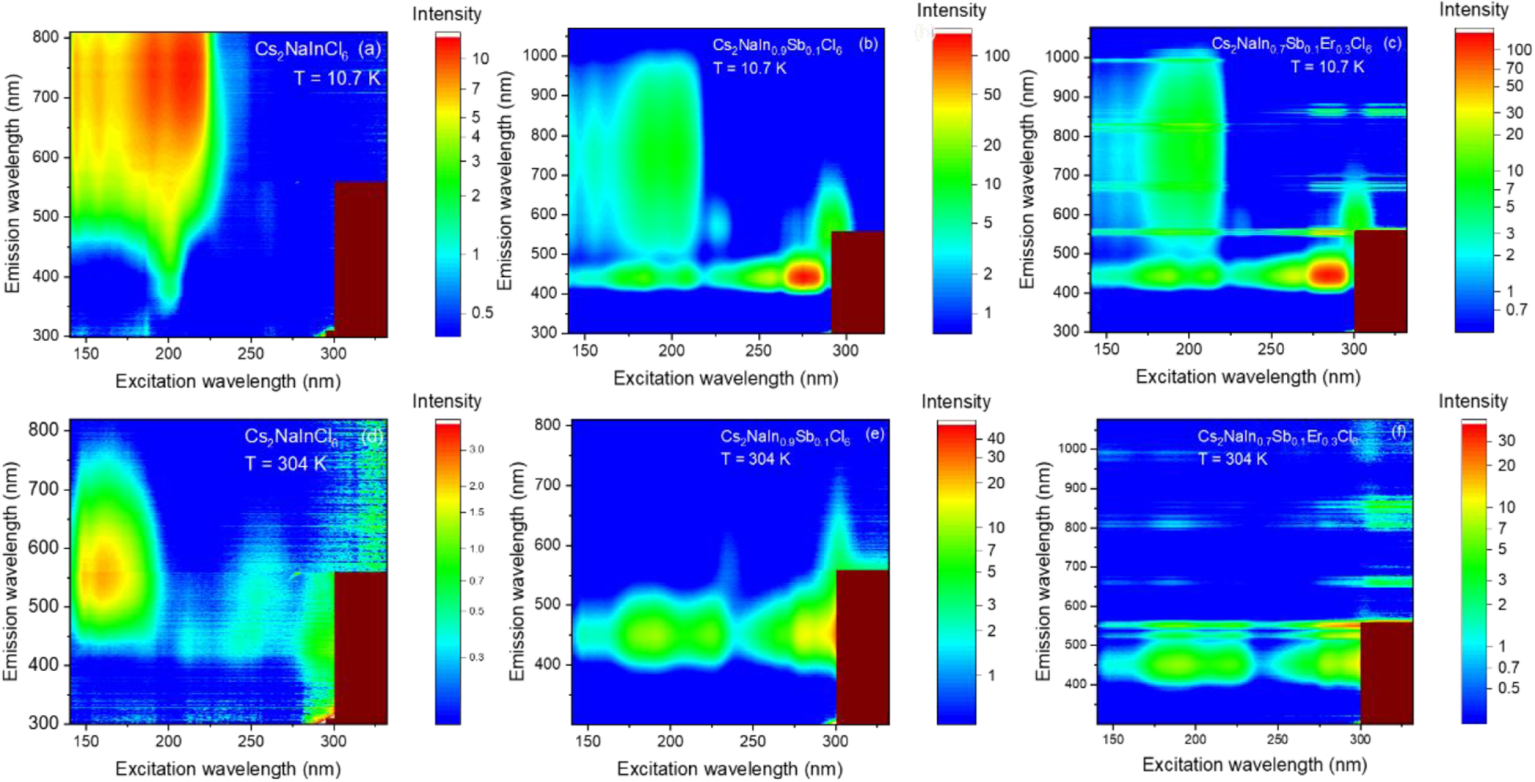
2D PLE maps measured at 10.7 and 304 K for the undoped, Sb-doped, and codoped with Sb and Er CNIC tested samples. Note that the intensities are represented on a logarithmic scale, Figure 6a,d, and e have an emission range only up to 810 nm.

The intensity of the “white” luminescence depended on temperature, excitation wavelength, and dopants. As the temperature increased to 304 K, the PL intensity of all samples decreased, and the “white” luminescence vanished, leaving only a 560 nm band in the PL spectrum of the CNIC. Additionally, a very weak band appeared at 450 nm with excitation at approximately 210, 250, and 320 nm. The excitation spectra of the CNIC (Figure 7a) show a dominant 186 nm band with weaker bands at 183, 174, 149, and 126 nm. We can conclude that this structure corresponds to the excitonic excitation spectrum, with the sharp peak at 186 nm attributed to a free exciton. Since this characteristic band structure vanished upon heating, we assume that the optical bandgap of the CNIC is equal to or greater than 6.7 eV, contrasting with the typical CNIC bandgap value reported in literature.^7–9^

**Figure 7:**
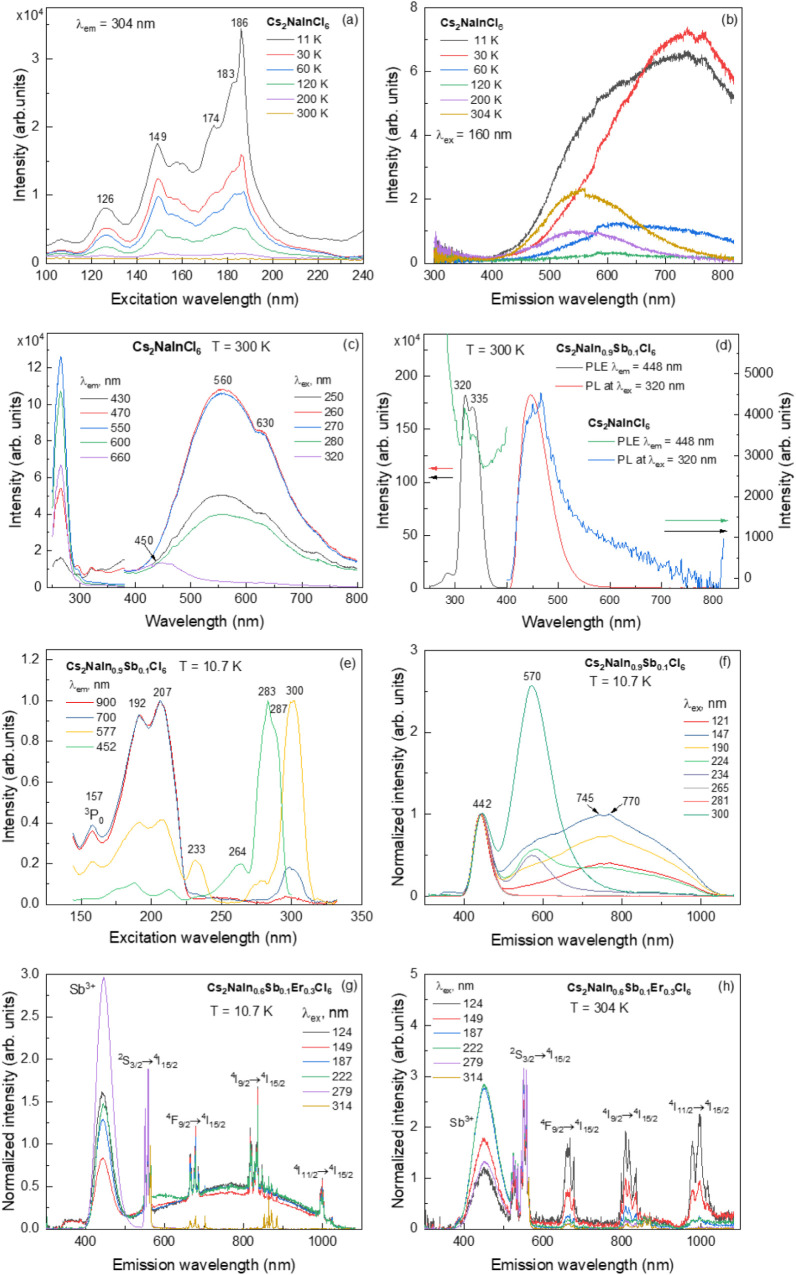
PLE and PL spectra of the undoped, Sb-doped, and codoped with Sb and Er CNIC samples.

Figure 7b shows the luminescence spectra of the CNIC sample excited at 160 nm and measured at a wide temperature range (11–304 K). Several peaks can be seen at wavelengths of 450, 564, 653, and 770 nm at different temperatures, each exhibiting varying intensities. For example, the band at 770 nm is only present below 30 K, while the band at 450 nm can be detected at 200 K. The intensity of the luminescence band at 653 nm (1.9 eV) decreases as the temperature becomes higher and is undetectable above 120 K, while the 564 nm band (2.2 eV) is more pronounced at temperatures above 120 K. By analyzing the complex relationship between the intensities of the 450, 564, 653, and 770 nm bands and temperature (Figures S3 and S4), we propose that this variation suggests different underlying mechanisms for each band, which are discussed below in the “Optical Spectrum Formation Mechanism” section.

The broadband emission of CNIC observed at 300 K under a 265 nm excitation localizes within the 400–800 nm range. It can be deconvoluted into three bands at approximately 450, 568, and 652 nm (Figure 7c), aligning with the broadband “white” emission reported for halide double perovskites in ref 23.

The excitation spectra of CNIC and Sb-doped CNIC at λ_em_ = 448 nm are similar (Figure 7d), but their intensities vary significantly, with the Sb-doped CNIC being over 30 times more intense. The presence of double peaks at 320 and 335 nm suggests the specific underlying mechanism driving their spectral features. Figure 7e,f displays the normalized PLE and PL spectra of the Sb-doped CNIC sample measured at 10.7 K. The PL spectra in Figure 7f consist of two relatively narrow bands at 442 and 570 nm, along with a broad band of “white” luminescence. The 442 nm peak is present in all the spectra and is excited by almost any light with a wavelength shorter than 300 nm. The similarity in the shape of the peak shoulder across all spectra, as well as the gradual rise on the opposite side, indicates the presence of at least two bands constituting this peak.

From the obtained results (Figure 7a–f), we can conclude that Sb doping reduces the bandgap by creating doping levels near the bottom of the conduction band, because the Sb-doped CNIC corresponds to ∼192 nm excitation wavelength (Figure 7e), which is longer than 186 nm excitation of the CNIC (Figure 7a). The 157 nm band observed in the PLE short-wavelength region of Sb-doped CNIC (Figure 7e) is related to the Sb^3+^ multiplet structure. Additionally, two bands at 192 and 207 nm, excited at monitoring wavelengths of 700 and 900 nm respectively, produce “white” luminescence in the 400–1050 nm range (Figure 7f). The 452 and 577 nm bands are only excited in the part of the spectrum with λ_ex_ > 225 nm. The signals in the λ_ex_ < 225 nm region (Figure 7e), obtained from the 452 and 577 nm emissions, result from the overlap with bands generating “white” luminescence. The high-intensity maximum, consisting of at least two bands at 283 and 287 nm under λ_em_ = 452 nm (Figure 7e), can be associated with the presence of Sb^3+^ ions within the crystal structure.

Figure 7g,h presents the PL spectra of the CNIC sample doped with Sb and Er at 10.7 and 304 K, respectively. The spectra reveal the characteristic multiplet structure of the intraionic transitions in Er^3+^ and the emission transitions in the Sb^3+^ ions. The spectra in Figure 7g,h is normalized at 564 nm, which corresponds to the Er^3+^ intraionic ^2^S_3/2_ → ^4^I_15/2_ transition.^24,25^ When excited with wavelengths longer than 222 nm, there is no “white” luminescence, nor any ^4^I_9/2_ → ^4^I_15/2_ and ^4^I_11/2_ → ^4^I_15/2_ transitions of the Er^3+^ ion at room temperature. At 10.7 K, Sb^3+^ luminescence is dominant, while Er^3+^ luminescence has more intense bands at room temperature.

The analysis of the optical spectra reveals several complex findings:

1 The absorption and excitation spectra of the CNIC and Sb-doped CNIC samples are similar, with only slight variations;
2 The CNIC has a larger bandgap than previously considered;
3 The intricate temperature evolution of the PLE and PL spectra cannot be explained by the STE transitions only.

In the following sections, we provide our explanations about the optical peculiarities of the CNIC and doped samples.

### Optical Spectrum Formation Mechanism

Based on the XPS results, which have revealed the presence of different charge states of the indium ions, we suggest that the optical spectra of the CNIC sample result from the appearance of optically active In^+^ ions, which have a 5s^2^ configuration. Nevertheless, the XPS results in Table 2 show that a small number of In^+^ ions is formed in the CNIC, 0.3 at. % or 1 × 10^20^ cm^–3^ and this amount enables detection of their optical response. Furthermore, it is reasonable to suggest that some In^+^ ions occupy Na^+^ positions during synthesis, as M^+^ type ns^2^ ions are known to be the primary luminescence centers in alkali halides with small amounts of ns^2^ ions.^26–28^ Since the electron configurations of In^+^ and Sb^3+^ are identical (5s^2^), the optical spectra of undoped CNIC resemble Sb-doped CNIC spectra (Figure 7d).

The XPS data indicate that the samples doped with Sb contain not only Sb^3+^ ions, but also Sb^5+^ ions with electron configuration 4d,^10^ which is optically inactive. Additionally, the Sb-doped CNIC contains In^+^ and In^3+^ ions (see Tables 2, S1–S3 and Figures 2, 4). Therefore, the undoped CNIC shows the low-intensity luminescence caused by the presence of a small number of In^+^ ions, and the Sb-doped CNIC shows the integral luminescence formed by Sb^3+^ and In^+^ active ions with a 5s^2^ electron configuration. However, we cannot rule out that other mechanisms contribute to the optical spectra.

To verify our assumption, we analyzed and separated various components of the absorption spectra of the undoped and Sb-doped CNIC (Figure 5) for closer examination. To reveal individual components under conditions of minimal bands and arbitrary bandwidths, we separated the overlapped features and uncovered a complex multiplet structure associated with the existence of the 5s^2^ ions in both cases (Figure 8 a,b).

**Figure 8:**
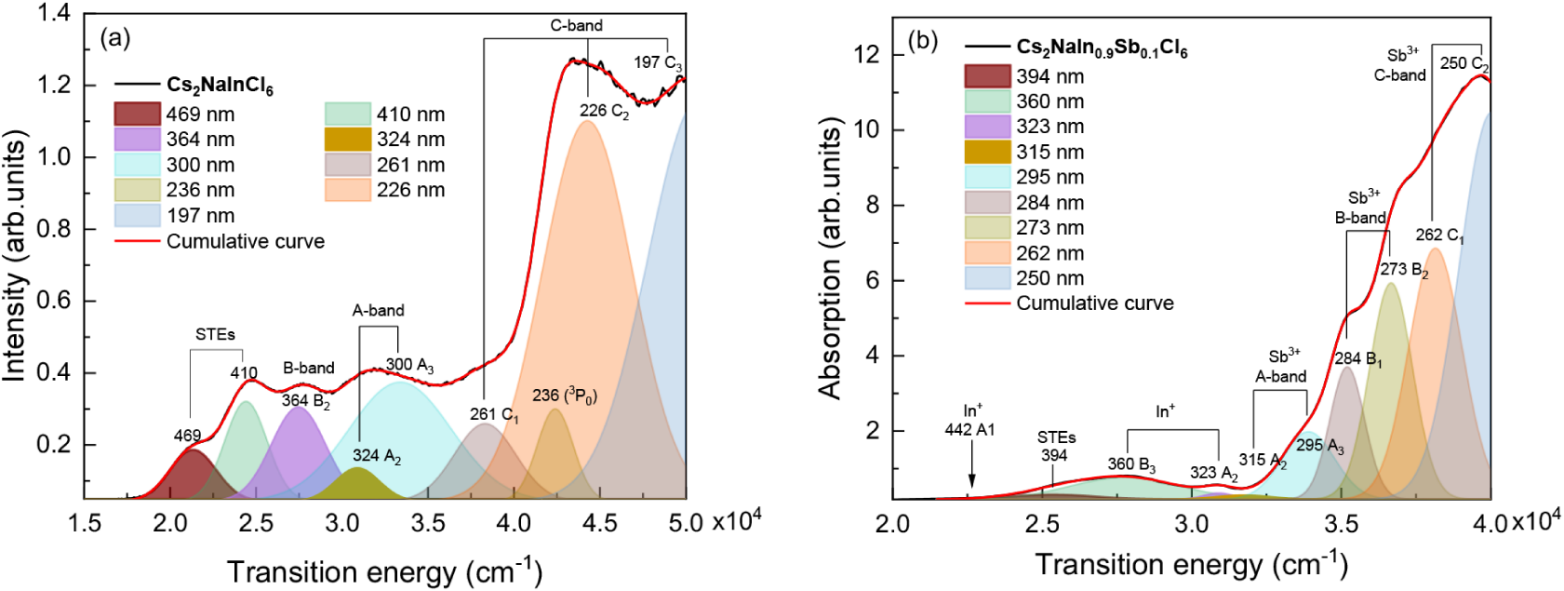
Deconvolution of the absorption spectra of the CNIC (a) and Sb-doped CNIC (b) samples.

The energy structure of a free ns^2^ ion and the ns^2^ ion in an octahedral crystal field is well-known and described in refs 29–33 . The ground state of the free 5s^2^ ion is ^1^S_0_ and the excited states are ^3^P_0_, ^3^P_1_, ^3^P_2_, and ^1^P_1_, corresponding to the 5s^1^p^1^ electron configuration. Due to the selection rules, the transitions between the ground ^1^S_0_ state and the excited ^3^P_0_, ^3^P_1_, and ^3^P_2_ terms are spin-forbidden. However, the strong spin–orbit interaction mixes the ^3^P_1_ and ^1^P_1_ terms with the same total angular momentum (J), thereby removing these restrictions. In this case, the ^1^S_0_ ↔ ^3^P_0,1,2_ transitions become partially permitted and can be observed in optical spectra.

In the regular [(Sb/In)­Cl_6_] octahedron with *O*_*h*_ position symmetry, the fifth-fold degenerated ^3^P_2_ term splits into a triplet T_2u_ and doublet E_u_. The other two terms represent unsplit triplets: ^3^P_1_ (T_1u_) and ^1^P_1_ (T_1u_). Thus, under *O*_*h*_ symmetry, only three intensive bands can be observed in optics: the ^1^S_0_ → ^3^T_1u_ transition (A-band), ^1^S_0_ → ^3^T_2u_ transition (B-band), and ^1^S_0_ → ^1^T_1u_ transition (C-band). However, the absorption spectra in Figure 8 show more than three bands, indicating that there are low-symmetry distortions in the octahedral [(Sb/In)­Cl_6_] coordination complexes. These distortions can specifically be Jahn–Teller (JT) distortions of two types: *Q*_2_ and *Q*_3_.^34^ In the case of only the *Q*_2_-type JT distortions, reducing the *O*_*h*_ site symmetry to *C*_*4v*_, each triplet splits into two terms: ^1^T_1u_ → ^1^A_2u_ + ^1^E_u_ (C-band), ^3^T_1u_ → ^3^A_2u_ + ^3^E_u_ (B-band), and ^3^T_2u_ → ^3^A_1u_ + ^3^E_u_ (A-band).^33,34^ Thus, six transitions are possible and must be detected in the absorption spectrum. When either the *Q*_3_-type distortions are present or both types of JT distortions act together, the site symmetry reduces to *C*_*2v*_. In this scenario, each triplet splits into three singlets, resulting in the absorption spectra potentially exhibiting up to nine bands. The existence of such a complex multiplet structure has been proven theoretically in refs 33,34 and this type of multiplet structure is observed in the absorption spectrum (Figure 8).

In the CNIC sample, ^3^P_0_ can be excited using a 236 nm wavelength, as seen in Figure 8a of the absorption spectrum. It should be noted that the ^3^P_0_ singlet does not interact with the crystal field and remains unchanged. In the Sb-doped CNIC, ^3^P_0_ can be excited with a wavelength of 157 nm. Although it is absent from the absorption spectrum in Figure 8b, it is visible in the excitation spectrum in Figure 7e. The transitions at 469, 410 (Figure 8a) and 394 nm (Figure 8b) can be assigned to the STE transitions within the CNIC and Sb-doped CNIC matrix, respectively.

The absorption bands at 324 (A_2_) and 300 (A_3_) nm in Figure 8a, as well as the PL band at 450 (A_1_) nm in Figure 7c, can be attributed to the A-bands of In^+^. This aligns with ref 35 in which CsI doped with In^+^ exhibits PL bands at 2.22 eV (558 nm) and 2.49 eV (497 nm) with 304 nm excitation. A similar A_2_ band originating from the In^+^ ions appears at 323 nm in the absorption spectra of the Sb-doped CNIC (Figure 8b). Furthermore, the Sb-doped sample exhibits two bands at 315 (A_2_) and 295 (A_3_) nm, similarly to A-bands of Sb^3+^. When excited with close wavelengths (320 nm), the Sb-doped CNIC emits at 442 (A_1_) nm (Figure 7d).

The 364 nm (3.4 eV) band seen in the absorption spectrum in Figure 8a is a part of the B-bands.^28,36^ The analysis of the temperature evolution of the PL band of the CNIC sample shows that the integral intensity of the 450 and 564 nm bands changes with the temperature. Therefore, these PL bands can also be attributed to the B-bands of In^+^ in the CNIC sample (Figure 7 b,c and Figures S3 and S4).

We identified three intense bands labeled C_1_, C_2_, and C_3_ at 261, 226, and 197 nm, respectively, as components of the C-bands in the CNIC sample (Figure 8a). In contrast, the Sb-doped sample shows only two C-band components centered at 262 (C_1_) and 250 (C_2_) nm (Figure 8b). It is known from the literature that for alkali halide phosphors doped with In^+^ ions, the C/A ratio is approximately 50.^33,34^ In our case for the CNIC sample, the C/A ratio is about 25; while for the Sb-doped sample, it equals 100.

### DFT Calculation Results

Methods of the DFT calculation are described in the Supporting Information. Geometry optimization of the cubic CNIC structure revealed that the lattice parameter changes slightly from *a* = 10.5195 Å to *a* = 10.3744 Å, with a deviation of about 1.34%. Thus, the selected computing method reproduces the geometric parameters of the CNIC crystal structure quite satisfactorily. Substitution of In^3+^ on Er^3+^ or Sb^3+^ increases the unit cell volume due to the larger ionic radii of Er^3+^ (0.89 Å) and Sb^3+^ (0.86 Å).^19,20^ However, it does not influence the host CNIC structure stability: total energy per atom equals 115.20 and 115.38 eV, for Er^3+^ and Sb^3+^-doped structure, respectively. Considering a preliminary ionic nature of the compound, the presence of Cl^–^, Na^+^, and In^3+^ vacancies (V) also increases the lattice parameters. The V_Na+_ and V_In3+_ decrease the total energy of CNIC, thereby stabilizing the structure, while the V_Cl^–^_ increases the total energy of the CNIC, destabilizing the structure (Table S4).

The lattice parameters calculated for various models of the CNIC structure with an excess of In^+^ are smaller than those for the ideal CNIC structure (Table S4). The appearance of Sb^5+^ ions create vacancies in the cation sublattice for charge compensation and reduces the lattice stress, leading to increased unit cell dimensions (Table S4). This can explain why the modeled structure (Cs_2_Na_0.97_(In^+^)_0.03_(In^3+^)_0.9_(Sb^3+/5+^)_0.05_□_0.05_Cl_6_) is highly stable.

The energy band structures were calculated using optimized crystallographic structures. The spin-up and spin-down calculations of the CNIC crystal indicated that it is a direct semiconductor with a 5.61 eV bandgap (Figure 9). It is important to note that DFT calculations typically underestimate the bandgap value. This fact explains the discrepancy between the experimental (6.7 eV) and the calculated value. The top of the valence band (VB) is formed by the Na 2p and Cl 3p electrons, and the conduction band (CB) is composed of In 5p electrons hybridized with the Cl 3p electrons. The Na band in the VB is localized, distinct, and flat; however, this has no significant effect on the electronic properties.

**Figure 9:**
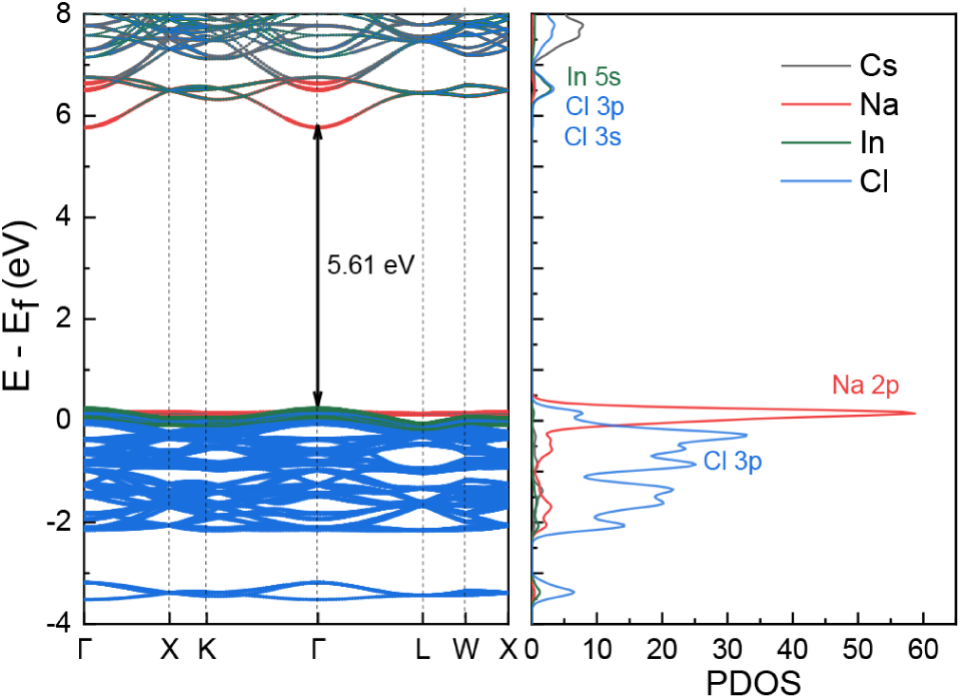
Energy band structure of the CNIC crystal.

The energy band structures of CNIC doped with Sb^3+^ were calculated using a spin-restricted approach (Figure 10). In this scenario, the hybridization of Sb 5p and Cl 3p electron states (spin-up) and Sb 5p and Cl 3s (spin-down) generates a donor trapping level between 3.60 and 3.85 eV, which notably reduces the forbidden zone and agrees with the measured absorption spectra (Figure 5). For this reason, Sb^3+^ doping could remarkably improve PL performance by breaking the selection rules for parity-forbidden transitions.^10^

**Figure 10:**
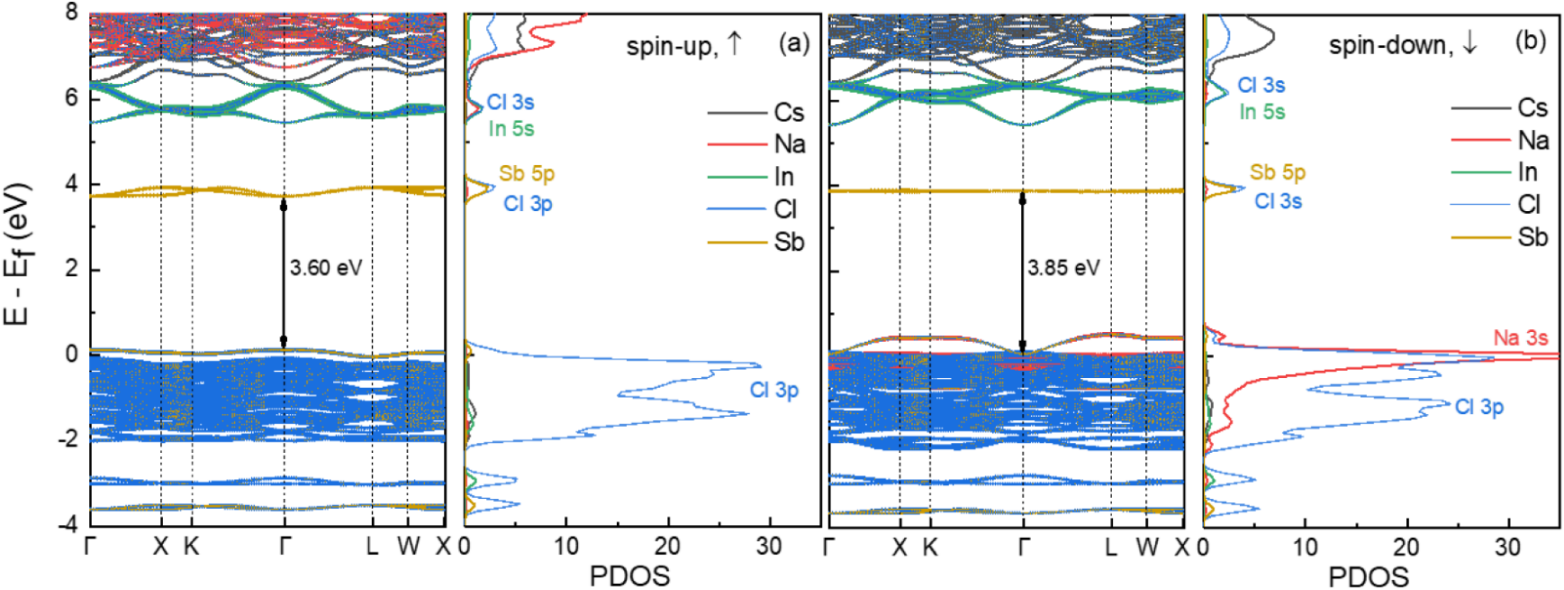
Energy band structures of the CNIC crystals doped with Sb^3+^ calculated under a spin-restricted polarization (spin-up ↑ and spin-down ↓): (a) Sb-doped CNIC (↑); (b) Sb-doped CNIC (↓).

The energy band structures shown in Figure 11 were calculated for CNIC crystals containing V_Cl^–^_, V_Na^+^_, and V_In3+_ vacancies. As seen in Figure 11a and b, V_Cl^–^_ vacancies create donor trapping levels near 3.63 and 3.65 eV and they are mainly composed of In electrons. By contrast, V_Na^+^_ vacancies generate donor trapping levels at approximately 4.01 eV and consist primarily of Na electrons (Figure 11d). Forming this way, the vacancy-originated sublevels decrease the energy bandgap of CNIC, enabling additional optical transitions in the CNIC crystals. High-energy optical measurements conducted at DESY in Hamburg, Germany, have shown that the CNIC bandgap may be considerably larger than previously believed. This finding clarifies the narrower bandgap observed earlier, which can be explained by the sub-bands within the forbidden zone caused by crystal defects.

**Figure 11:**
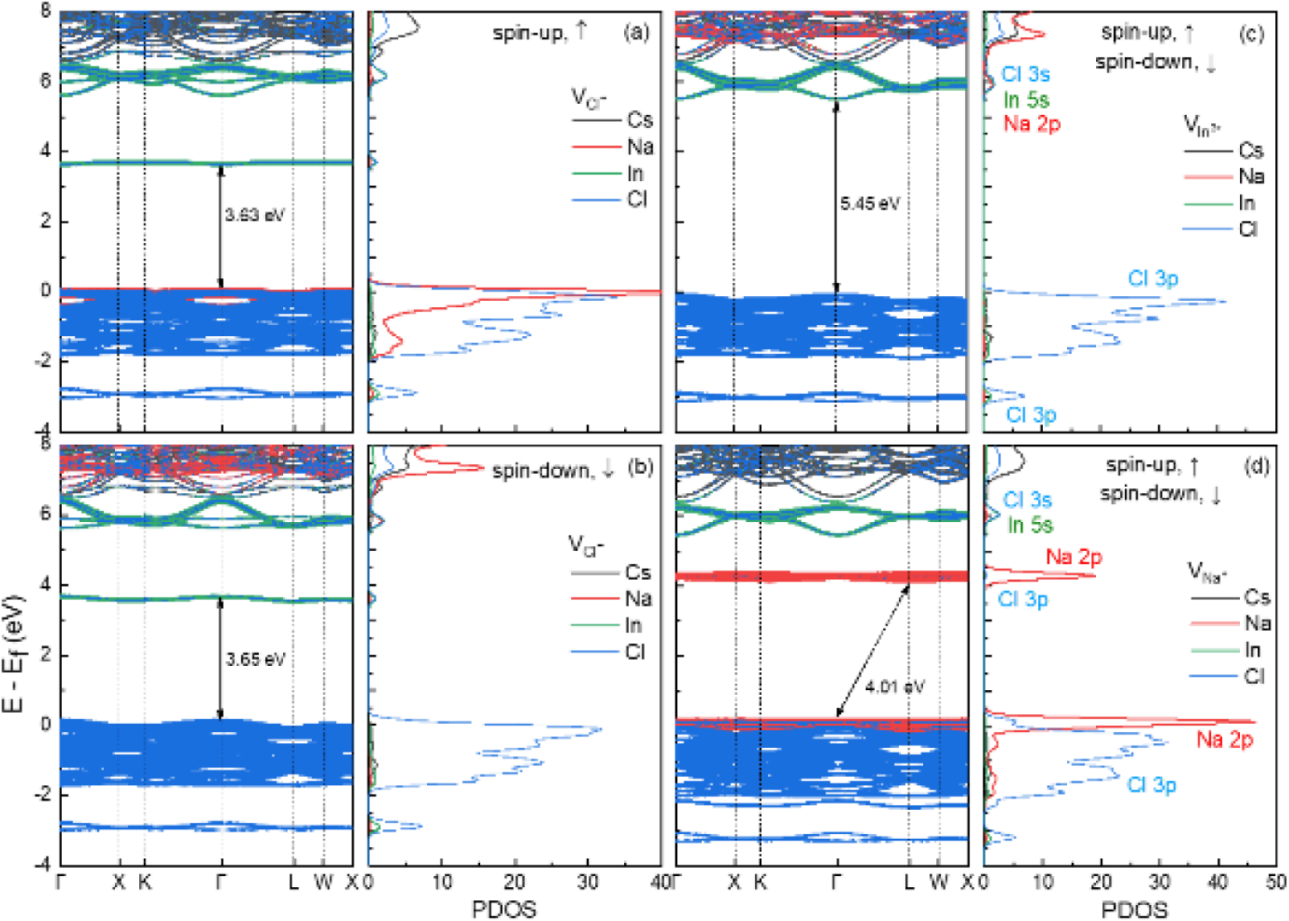
Energy band structures of the CNIC with vacancies calculated under a spin-restricted polarization (spin-up ↑ and spin-down ↓): (a) CNIC with V_Cl^–^_ (↑); (b) CNIC with V_Cl^–^_ (↓); (c) CNIC with V_Na_^+^ (↑ and ↓); (d) CNIC with V_In_^3+^ (↑ and ↓).

The crucial questions to be answered concern the mechanism behind the formation of the In^+^ ions in the CNIC sample and how charge transfer occurs. We hypothesize that the deficiency of Na^+^ ions in the CNIC composition (Table 2) results in a replacement of Na^+^ by In^+^ ions, which form due to the charge transfer from Cl^–^ ions to In^3+^ ions. To support this assumption, the energy band structure of the CNIC crystal with a deficiency of In and an excess of Na ions (Figure 12a) was calculated. Figure 12b,c,d shows the energy band structure of CNIC crystals with the deficiency of Na and an excess of In ions.

**Figure 12:**
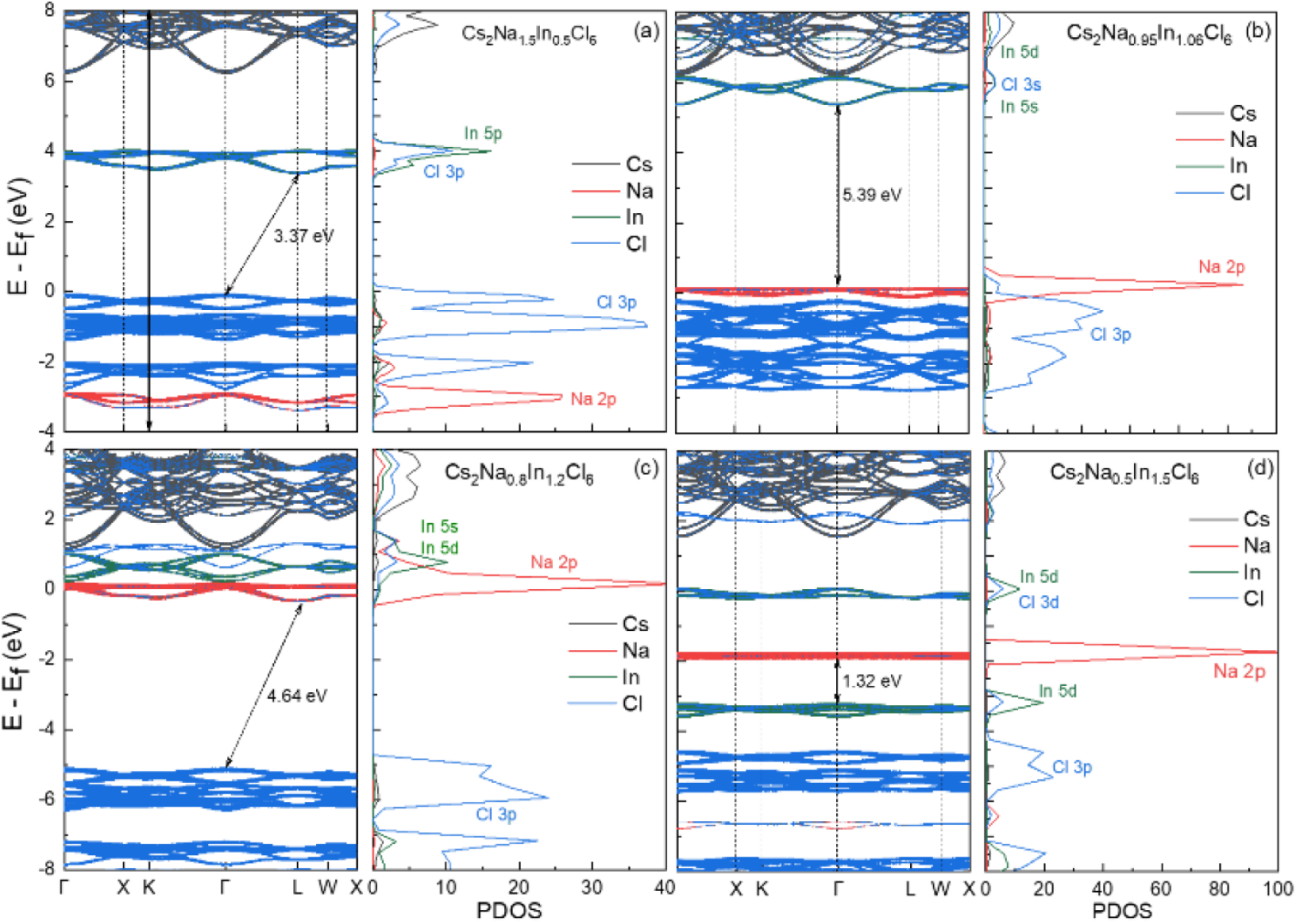
Energy band structures calculated for crystals with nonstoichiometric content: (a) Cs_2_Na_1.5_In_0.5_Cl_6_; (b) Cs_2_Na_0.94_In_1.06_Cl_6_; (c) Cs_2_Na_0.8_In_1.2_Cl_6_; (d) Cs_2_Na_0.5_In_1.5_Cl_6_.

The obtained band structures were compared with the stoichiometric undoped CNIC structure illustrated in Figure 9. For In-deficient CNIC, the split-off band composed of Cl 3p and In 5p electrons shifts down by up to 3.37 eV (Figure 12a). Increasing the In ions to Na ions gradually restructures the crystal band structure, e.g., a slight increase of the In ions up to Cs_2_Na_0.94_In_1.06_Cl_6_ insignificantly decreases the bandgap to 5.39 eV (Figure 12b). Further increase of In up to Cs_2_Na_0.8_In_1.2_Cl_6_ results in the appearance of the donor band, which fills with the In electrons and gets narrower and denser. At the same time, the energy band formed by the Na ions shifts up to the CB and indirect transitions are permitted (4.64 eV) (Figure 12c). The following increase of the In content to Cs_2_Na_0.5_In_1.5_Cl_6_ fully rearranges the VB and CB, which exfoliates and shifts down, decreasing the bandgap to 1.32 eV (Figure 12d). There is an excess of electron density on In along with a noticeable depletion of electron density on both Na and Cl. Electrons then transfer from Na and Cl ions to an In ion, and the electron-rich In centers suggest the possibility of self-trapping. This model has a significant charge imbalance, substantial polarization, and pronounced effects on conductivity and optical absorption due to deep states.

In Figure 13, the electron density difference between stoichiometric and nonstoichiometric Cs_2_Na_1–x_In_1+x_Cl_6_, x = 0.06, 0.20, 0.50, is presented. The electron density of the CNIC crystal represents a reference distribution where the charge configuration is balanced. In the Cs_2_Na_0.94_In_1.06_Cl_6_ structure (Figure 13a), electrons transfer from Cl^–^ ions to In^3+^ ions, creating local charge inhomogeneity and resulting in an increase of the electron density on the In^3+^ ions (brown circles). This suggests a donor behavior of Cl, donating charge to In, indicating the band tailing or defect states. As the excess of In grows, its states become denser, increasing localization of electrons on In and creating donor-like states with more pronounced charge polarization around Cl^–^ (Figure 13b,c). Finally, an excess of electrons is observed on In, and a deficiency is observed near Na, and the system starts forming localized states, or small polarons.

**Figure 13:**
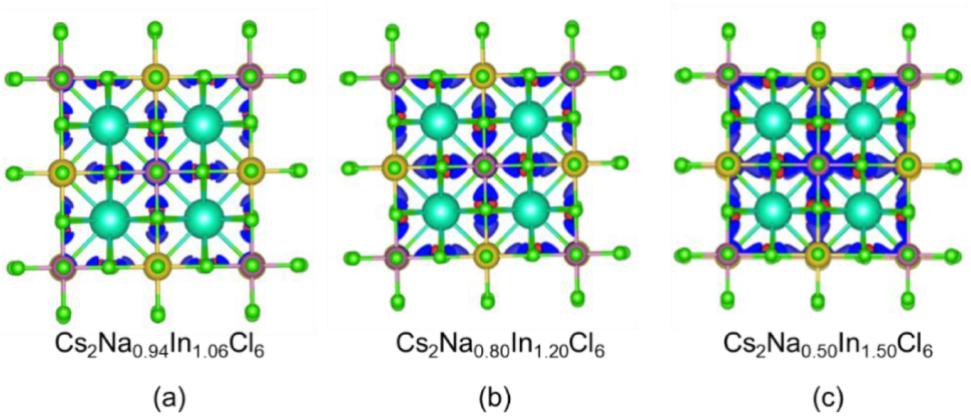
Electron density distribution in Cs_2_Na_0.94_In_1.06_Cl_6_, Cs_2_Na_0.8_In_1.2_Cl_6_, and Cs_2_Na_0.5_In_1.5_Cl_6_ model structures. The composition ions are colored as follows: In—brown; Na—orange; Cs—emerald; Cl—green.

This explanation of the DFT calculation results supports the idea of electron transfer to In^3+^, followed by In^+^ formation. Cl^–^ changes the charge to Cl^0^ and dimerizes with an adjacent Cl^–^ to form the dimer Cl_2_^–^.^37^

Figure 14 shows the calculated energy band structure of the modeled Cs_2_Na_0.97_(In^+^)_0.03_(In^3+^)_0.9_(Sb^3+/5+^)_0.05_□_0.05_Cl_6_ crystal. According to Figure 10, the Sb^3+^ ions have strongly localized 5p states that form a narrow energy band in the midgap position. Figure 14 depicts the likely formation of Sb^5+^ cations that create hole traps. Incorporating Sb^3+^ as a dopant into the CNIC sample introduces a deep trap level located approximately in the middle of the bandgap. This trap originates from the electronic states of antimony, which form localized energy levels within the forbidden energy range. Because of its midgap position, the Sb-induced trap can act as a recombination center, capturing both electrons and holes. Depending on the recombination dynamics, the trap can either enhance PL by enabling radiative recombination (e.g., broadband emission via self-trapped excitons) or suppress carrier transport and contribute to nonradiative losses if the recombination is nonemissive. Overall, the Sb-induced trap plays a crucial role in adjusting the material electronic and optical properties, affecting its luminescence efficiency, carrier lifetime, and potential applications in scintillation, lighting, and charge storage.

**Figure 14:**
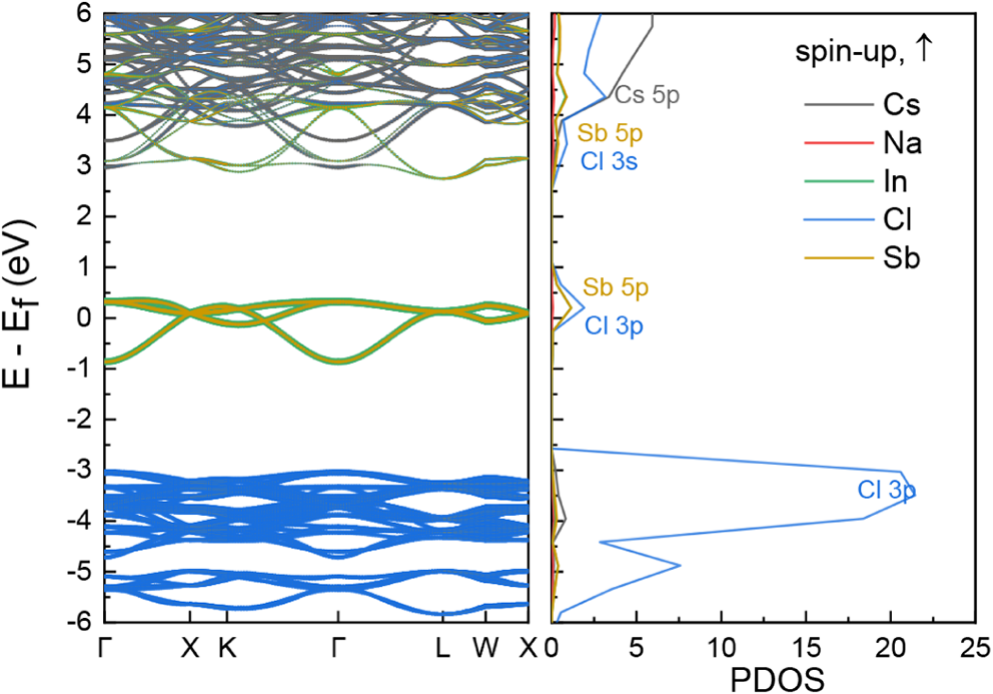
Energy band structure of the Cs_2_Na_0.97_(In^+^)_0.03_(In^3+^)_0.9_(Sb^3+/5+^)_0.05_□_0.05_Cl_6_ crystal under a spin-restricted polarization (spin-up ↑ is presented).

Based on our analysis of the absorption spectra (Figure 8) and the DFT results, we composed the energy level splitting scheme for the In^+^ and Sb^3+^ ions (Figure 15). The scheme includes the energy levels of the free In^+^ and Sb^3+^ ions taken from the NIST database,^16,17^ the energy levels for In^+^ and Sb^3+^ in the CNIC crystal matrix extracted from the absorption spectra, and the trapping levels predicted by the DFT calculations.

**Figure 15:**
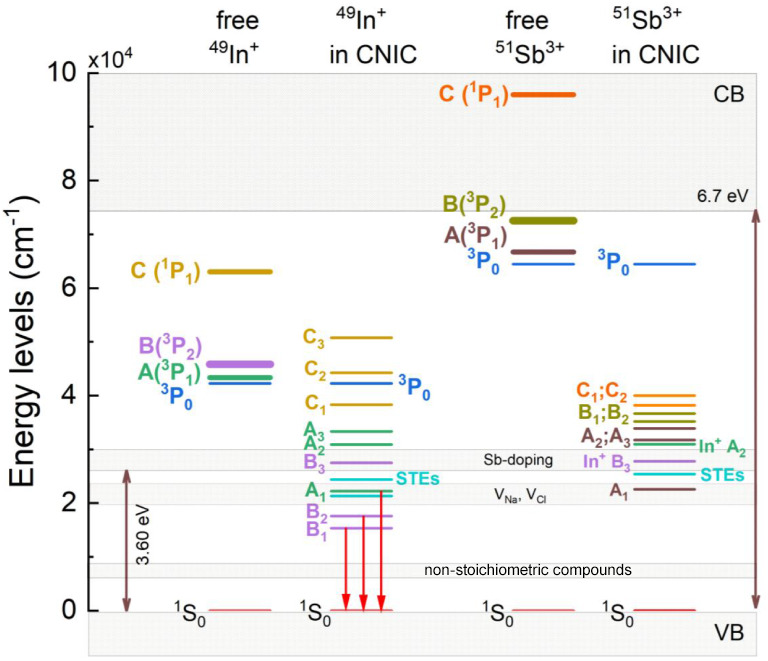
Energy level splitting scheme of the Sb^3+^ and In^+^ ions, free and placed in the CNIC matrix.

One can see that In^+^ and Sb^3+^ have the same electron configurations, but slightly different nuclear charges (^49^In and ^51^Sb) that lead to a different arrangement of the free indium and antimony energy levels.^16,17^ The CNIC crystal field splits the ^3^P_2_, ^3^P_1_, and ^1^P_1_ terms into nine singlets according to the *C*_2*v*_ symmetry. Because the 5s^2^ electron configuration interacts directly with the chlorine crystal field, the energy level splitting is expected to be significant. The ^3^P_0_ term is a singlet with J = 0 and it does not interact with the crystal field, remaining in its original position. The largest splitting occurs in the ^3^P_2_ term, because J = 2 is the maximum total angular momentum.

A comparison of the CNIC and Sb-doped absorption spectra (Figures 5 and 8) demonstrates that the crystal field splitting of the In^+^ ions is greater than that of the Sb^3+^ ions. It could be explained that replacing In^3+^ with Sb^3+^ ions in the CNIC matrix does not significantly excite the [SbCl_6_] coordination complex because of the small difference in ionic radii and the high symmetry of their electron configurations. In contrast, incorporating In^+^ in the Na^+^ positions strongly disturbs the crystal field because the difference in their ionic radii exceeds 50%. Moreover, the nearest Cl^–^ vacancies and the resulting charge transfer reduce the In^+^ site symmetry. Therefore, we can conclude that the [SbCl_6_] coordination complex undergoes the JT distortions of the *Q*_2_ type, while the In^+^ ion experiences the JT distortions of the *Q*_3_-type or their linear combination.

The trapping levels resulting from In^+^/In^3+^ cation disorder and the related charge transfer from Cl^–^ ions to In^3+^, combined with Sb^3+^ doping and other defects in the CNIC structure mentioned earlier, are illustrated in Figure 15. These factors produce broad bands that represent calculation errors, leading to the formation of a complex energy structure within the forbidden zone.

## Conclusions

We have proposed a theoretically justified, and experimentally confirmed mechanism for the formation of the optical spectrum in Cs_2_NaInCl_6_, which is doped with Sb and Er. The significant results were achieved through high-energy optical measurements conducted at the P66 time-resolved VUV spectroscopy beamline at the PETRA III storage ring in DESY, Hamburg, Germany. Based on the PL spectra and DFT bandgap calculations, we conclude that the CNIC bandgap is considerably larger than previously believed: 6.7 eV instead of 5.1 eV. This finding clarifies the narrower bandgap observed earlier, which can be explained by the sub-bands within the forbidden zone caused by crystal defects. Additionally, we have found that the bandgap of the Sb-doped sample is smaller and corresponds to approximately 192 nm of excitation wavelength. Therefore, the Sb doping results in a reduced bandgap by forming doping levels near the bottom of the conduction band. Lastly, we have identified the ^3^P_0_ term in the Sb-doped CNIC sample, which had not been observed earlier. The ^3^P_0_ term is visible in the excitation spectrum at about 157 nm wavelength in Figure 7e.

We have found that the deficiency of Na^+^ and Cl^–^ ions is the main factor that determines the optical properties of CNIC-based compounds. The precipitation technique causes the formation of Na^+^ and Cl^–^ vacancies, resulting in two optical mechanisms. The first is related to the formation of self-trapped excitons based on V_Na^+^_ and V_Cl^–^_ vacancies, which create traps in the forbidden zone, contributing to “white” luminescence.

The second mechanism involves the formation of ns^2^ ions as additional optical centers as a result of the following redox processes: In^3+^ + 2e^–^ → In^+^. The In^3+^ ions changing their valence states to +1 occupy Na^+^ positions and create cation disorder in CNIC-based samples. The formation of In^+^ is accompanied by the shifting of electron density from Cl^–^ to In^3+^, resulting from the lack of Na^+^, and ultimately, an increase in In^+^ ions in the CNIC-based samples. The incorporation of the In^+^ into the Na^+^ positions strongly disturbs the surrounding crystal field and reduces the In^+^ site symmetry. It is explained by the substantial difference in the ionic radii between Na^+^ and In^+^, which exceeds 50%, as well as the charge transfer from the nearest Cl^–^ vacancies.

Thus, the CNIC-based luminescence spectrum consists of the PL luminescence caused by the STEs and the intraionic luminescence from the ns^2^ ions, which are interconnected. A lack of Na^+^ ions triggers the formation of both In^+^ ions and vacancy traps, which initiate the STE mechanism.

Embedding Sb^3+^ into the CNIC matrix and the appearance of Sb^5+^ ions does not significantly excite the [SbCl_6_] coordination complex because of the small difference in ionic radii and the high symmetry 5s^2^ electron configuration. However, it results in a crucial decrease in the total energy from 5787 eV for CNIC to 20.55 eV (see Table S4) for Cs_2_Na_0.97_(In^+^)_0.03_(In^3+^)_0.9_(Sb^3+/5+^)_0.05_□_0.05_Cl_6_, thereby increasing the probability of the following reactions:

$${\mathrm{In}}^{3+}+2e\to {\mathrm{In}}^{+};\;{\mathrm{Sb}}^{3+}+2h^{+}\to {\mathrm{Sb}}^{5+}$$

The codoping of Er^3+^ and Sb^3+^ cations into the CNIC crystal matrix significantly alters the coordination complexes [ErCl_6_] and [SbCl_6_], leading to a reduction in crystal symmetry from cubic to tetragonal. In this scenario, the PL spectra reveal the multiplet structure of the intraionic transitions of Er^3+^ ions, along with the emission transitions of Sb^3+^ ions, the 5s^2^ dopant. As a result, the [SbCl_6_] coordination complex experiences JT distortions of the *Q*_2_ type, while In^+^ ions undergo JT distortions of the *Q*_3_ type, or a combination of both.

## Supplementary Material



## Notes

XPS analysis description; EDS and XRD measurements; optical parameter measurements; Table S1: Alternative results of crystal structure refinement of Cs_2_NaInCl_6_ and Cs_2_NaInCl_6_ doped with 1 at. % Sb, considering changes in oxidation states of In^3+^ and Sb^3+^; Table S2: Refined atomic coordinates and isotropic thermal parameters in various structural models; Table S3: Interatomic distances and coordination numbers of atoms in calculated structural models; Table S4: Lattice parameters and total energy of atomic systems based on the CNIC crystal structure; Figure S1: Photographs of powders and SEM images of microcrystals with compositions Cs_2_NaInCl_6_, Cs_2_NaIn_0_._9_Sb_0_._1_Cl_6_, and Cs_2_NaIn_0_._6_Er_0_._3_Sb_0_._1_Cl_6_; Figure S2: Elemental maps for Cs_2_NaIn_0_._6_Er_0_._3_Sb_0_._1_Cl_6_; Figure S3: Temperature dependence of CNIC PL band intensities; Figure S4: CNIC PL emission bands at different temperatures (PDF)
