# Supplementary material for: A Novel Mechanism of Optical Spectrum Formation in the Cs2NaInCl6 Doped with Sb and Er
Source: Chem Mater. 2026 May 28;38(11):5384–96. doi: 10.1021/acs.chemmater.5c03256 (PMC13255095; doi:10.1021/acs.chemmater.5c03256)
Supplement: Supplementary file 1 [file cm5c03256_si_001.pdf]

Supporting information for

A Novel Mechanism of Optical Spectrum Formation in the $Cs_2NaInCl_6$ Doped with Sb and Er

Inna A. Ivashchenko[1], Małgorzata Makowska-Janusik[2], Lubomir D. Gulay[3], Yurij G. Kazarinov[4], Karina V. Lamonova[5], Yevheniia Smortsova[6], Anatoli I. Popov[7], and Katarzyna Matras-Postołek[*1]

[1]*Faculty of Chemical Engineering and Technology, Cracow University of Technology, Warszawska St. 24, 31-155 Cracow, Poland*

[2]*Faculty of Science and Technology, Jan Dlugosz University, Al. Armii Krajowej 13/15,42-200 Czestochowa, Poland*

[3]*Lesya Ukrainka Volyn National University, Voli Ave. 13, 43000 Lutsk, Ukraine*

[4]*National Science Center Kharkiv Institute of Physics and Technology, Akademichna St. 1, 61108 Kharkiv, Ukraine*

[5]*Donetsk Institute for Physics and Engineering named after O.O. Galkin, NAS of Ukraine, Nauky Ave. 46, 03028 Kyiv, Ukraine*

[6]*Deutsches ElektronenSynchrotron DESY, Notkestr. 85, 22607 Hamburg, Germany*

[7]*Institute of Solid State Physics, University of Latvia, Riga, LV-1063, Latvia*

[*]*Email: prof. K. Matras-Postołek (k.matras@pk.edu.pl)*

Table of Contents:

1. SEM analysis.

2. XPS analysis description.

3. EDS, XRD measurements.

4. Optical parameter measurements.

Table S1. Alternative results of crystal structure refinement of $Cs_2NaInCl_6$ and $Cs_2NaInCl_6$ doped with 1 at.% Sb, considering the change in oxidation states of In^{3+} and Sb^{3+}.

Table S2. Refined atomic coordinates and their isotropic thermal parameters in various structural models.

Table S3. Interatomic distances and coordination numbers (C.N.) of atoms in the calculated structural models.

Table S4. Lattice parameters and the total energy of the atom systems based on the CNIC crystal structure.

Fig. S1 Photos of the powders and SEM photos of the microcrystals with the following compositions: $Cs_2NaInCl_6$ (a), (b); $Cs_2NaIn_{0.9}Sb_{0.1}Cl_6$ (c), (d); $Cs_2NaIn_{0.6}Er_{0.3}Sb_{0.1}Cl_6$ (e), (f).

Fig. S2 Maps of the elements for $Cs_2NaIn_{0.6}Er_{0.3}Sb_{0.1}Cl_6$ (see Table 2).

Fig. S3 Dependence of the CNIC PL band intensities on the temperature.

Fig. S4 The CNIC PL emission bands obtained at different temperatures.

1. SEM analysis.

In Figure S1, the powders obtained from the samples $Cs_2NaInCl_6$, $Cs_2NaIn_{0.9}Sb_{0.1}Cl_6$, and $Cs_2NaIn_{0.6}Er_{0.3}Sb_{0.1}Cl_6$, along with their SEM images, are displayed. In Figure S2, the element maps for the $Cs_2NaIn_{0.6}Er_{0.3}Sb_{0.1}Cl_6$ powder are shown, and the atomic percentages of Cs, Na, In, Sb, Er, and Cl are listed in Table 2 of the article.

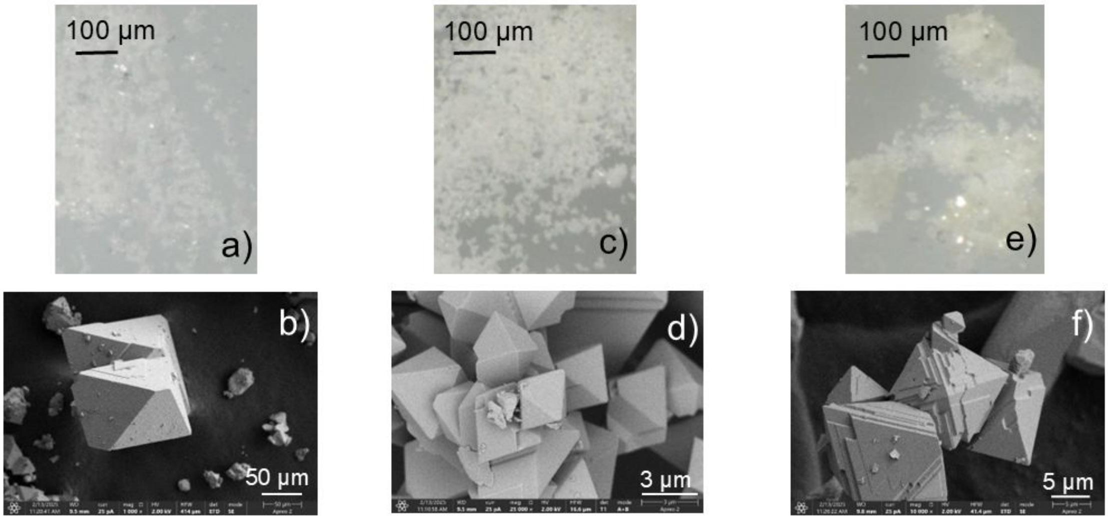

Fig. S1 Photos of the powders and SEM photos of the microcrystals with the following compositions: $Cs_2NaInCl_6$ (a), (b); $Cs_2NaIn_{0.9}Sb_{0.1}Cl_6$ (c), (d); $Cs_2NaIn_{0.6}Er_{0.3}Sb_{0.1}Cl_6$ (e), (f).

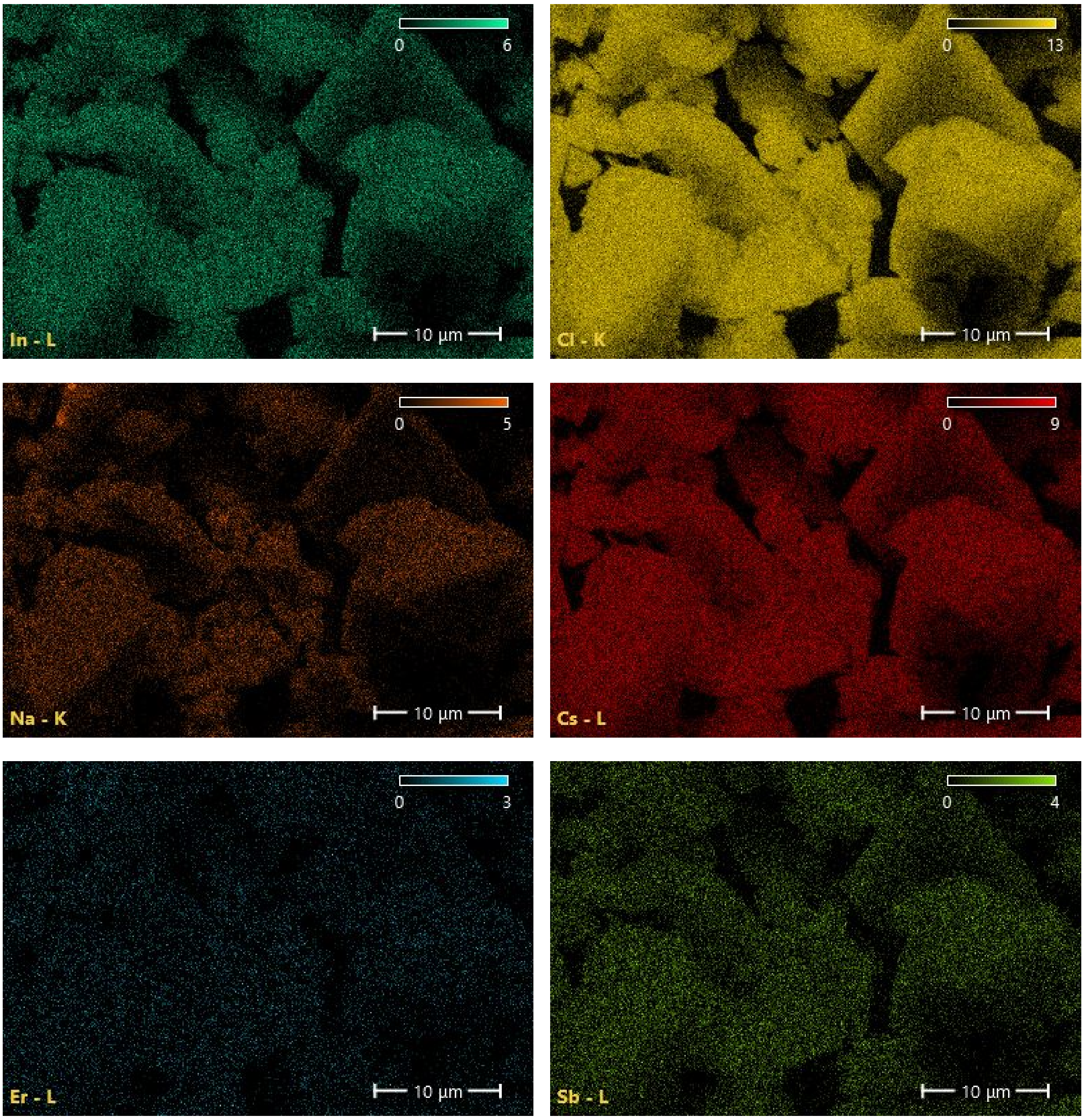

Fig. S2. Maps of the elements for $Cs_2NaIn_{0.6}Er_{0.3}Sb_{0.1}Cl_6$ (see Table 2).

2. XPS analysis description. The measurements were carried out in a PHI VersaProbeII Scanning XPS system using monochromatic Al Kα (1486.6 eV) X-rays focused to a 100 µm spot and scanned over the area of 400 µm^2. The photoelectron take-off angle was 45° and the pass energy in the analyzer was set to 117.50 eV (0.5 eV step) for survey scans and 46.95 eV (0.1 eV step) to obtain high-resolution spectra of C 1*s*, O 1*s*, Cl 2*p*, Na 1*s*, Er 4*d*, In 3*d*, Cs 3*d* and Sb 3*d* regions. A dual beam charge compensation with 7 eV Ar^+ ions and 1 eV electrons was used to maintain a constant sample surface potential regardless of the sample conductivity. All XPS spectra were charge referenced to the unfunctionalized, saturated C1*s* peak at 285.0 eV. The operating pressure in the analytical chamber was less than $4 \cdot 10^{-9}$ mbar. Deconvolution

of spectra was carried out using PHI MultiPak software (v.9.9.3). The Spectrum background was subtracted using the Shirley method. The information depth of the XPS analysis, within the geometry of the spectrometer, can be estimated at 5 nm.

3. EDS, XRD measurements. The element distribution was determined by means of energy-dispersive X-ray spectroscopy using an Apreo 2s LoVac (Thermo Fisher Scientific) scanning electron microscope. The obtained samples were investigated with X-ray powder diffraction (XRD) (using the SmartLab powder diffractometer, CuKα radiation, 2θ-range of 10-120°, 0.05° scan step). The crystal structures were calculated using the WinCSD software [1].

4. Optical parameter measurements. The absorption spectra were recorded using a Shimadzu UV-2600 UV-Vis spectrophotometer.

The PL properties within the VIS-NIR range were investigated using a Hitachi F-4600 fluorescence spectrometer at ambient temperature. According to the sample protocol, the powder was packed flat in a holder using tamping. The amount of powder used for measurement was determined by the number of mmol in each sample. We weighed the samples and, knowing their molar mass, calculated an equal mmol amount. This approach eliminated the dependence of spectral intensities on sample size.

PL emission (in the range 200–1100 nm) and excitation spectra (in the range of 100–332 nm) were obtained using the synchrotron radiation at the P66 time-resolved VUV spectroscopy beamline at the PETRA III storage ring of the Deutsches Elektronen-Synchrotron DESY (Hamburg, Germany). The excitation energy was set using a 2 m primary normal incidence McPherson monochromator with 0.8 nm resolution. The emission was analyzed using a 0.3 m Kymera 328i (Andor) spectrograph with the F/4.1 aperture and 0.4 nm spectral resolution. The detector was a thermoelectrically cooled CCD camera (Newton 920, BEX2-DD, Andor), and a set of high-pass Thorlabs filters were used to avoid the first and second orders in the data. The spectra were corrected both for the synchrotron radiation flux using the reference sodium salicilate excitation spectrum and for the sensitivity of the detection system using calibration curves produced within the previous measurements with Ocean Optics calibrated UV-VIS-NIR light source DH-2000. The automatic excitation emission matrix (EEM) scanning was performed using the home-made PyTango-based software with a GUI, recently available at P66. The measurements at low temperatures down to 10.7 K were performed using the ARS closed-cycle liquid helium-cooled cryostat, under 1.0×10^{-9} mbar ultra-high vacuum conditions.

5. DFT calculation. Quantum chemical calculations were conducted within the framework of density functional theory (DFT) as implemented in the Vienna Ab Initio Simulation Package (VASP). The projector augmented wave (PAW) method [2] was employed to describe the interaction between valence electrons and ionic cores. Electron exchange and correlation effects were treated using the Perdew-Burke-Ernzerhof (PBE) functional [3] within the generalized gradient approximation (GGA). The electronic configurations of all elements were modeled using spin-polarized sw-GW basis sets, appropriate for the treatment of open-shell systems and magnetic properties. An energy cutoff of 500 eV was used throughout the calculations to ensure sufficient convergence accuracy. The Brillouin zone was sampled using a 6 × 6 × 6 Γ-centered Monkhorst-Pack k-point grid for structural optimization tasks [4]. The structural relaxations were performed, allowing full relaxation of atomic positions, cell volume, and lattice shape. The convergence criteria were set to 10^{-5} eV for the total energy and 0.03 eV/Å for the atomic forces. To model the effect of dopant substitution, a series of supercells was generated in which Sb^{3+} ions were introduced into In sites, resulting in various In:Sb ratios. Representative dopant concentrations were selected to match the empirical data and to investigate the effect of varying doping levels on the host crystal structure and electronic properties. Each supercell was optimized, accounting for possible local distortions introduced by the Sb^{3+} dopant. The substitution was assumed to be isovalent, and charge neutrality was maintained in all configurations.

To analyze the electronic band structure, calculations were performed along a high-symmetry path in the Brillouin zone, defined as Γ–X–U–K–Γ–L–W–X, with the special points having the following coordinates: X (0.500; 0.000; 0.500), U (0.625; 0.250; 0.625), K (0.375; 0.375; 0.750), L (0.500; 0.500; 0.500), W (0.500; 0.250; 0.750). Spin polarization was included in all calculations, and Fermi-Dirac smearing was applied to improve convergence with a smearing width of 0.05 eV. For self-consistent field cycles, a convergence threshold of 10^{-8} eV was used to ensure high precision in total energy and charge density distribution.

Table S1. Alternative results of crystal structure refinement of $Cs_2NaInCl_6$ and $Cs_2NaInCl_6$ doped with 1 at.% Sb, considering the change in oxidation states of In^{3+} and Sb^{3+}

Empirical Formula	$\mathbf{Cs_2NaIn^{3+}Cl_6}$	$\mathbf{Cs_2Na_{0.97}In_{1.03}Cl_6}$<br>$Cs_2Na_{0.97}(In^{+})_{0.03}In^{3+}Cl_6$	$\mathbf{Cs_2NaIn^{3+}{}_{0.9}Sb^{3+}{}_{0.1}Cl_6}$	$\mathbf{Cs_2Na_{0.97}In_{0.93}Sb_{0.05}\square_{0.05}Cl_6}$<br>$Cs_2Na_{0.97}(In^{+})_{0.03}(In^{3+})_{0.9}$<br>$(Sb^{3+/5+})_{0.05}\square_{0.05}Cl_6$
Sp. Gr; Z;<br>Number of atoms in cell	$Fm\overline{3}m$; 4;<br>40	$Fm\overline{3}m$; 4;<br>40	$Fm\overline{3}m$; 4;<br>40	$Fm\overline{3}m$; 4;<br>39.8
Unit cell parameters, Å	a = 10.5359(2)	a = 10.5360(2) Å	a = 10.5398(3)	a = 10.5398(3) Å
V, Å^3	1169.52(7)	1169.56(7)	1170.82(9)	1170.8(1)
Calculated density (g/cm^3)	3.5001(2)	3.5155(2)	3.5001(3)	3.4806(3)
Absorption coefficient (1/cm)	770.46	775.05	772.42	767.53
Radiation and wavelenght (Å)	CuKα; 1.54185	CuKα; 1.54185	CuKα; 1.54185	CuKα; 1.54185
Diffractometer	SmartLab	SmartLab	SmartLab	SmartLab
Mode of refinement	Full profile	Full profile	Full profile	Full profile
Number of free parameters	6	7	7	7
R_I; R_p	0.0460; 0.2477	0.0447; 0.2441	0.0408; 0.2413	0.0410; 0.2321
Scale factor	0.5974(6)	0.5807(5)	0.5759(5)	0.5433(3)
Texture axis and parameter	[0 1 3] 0.94(3)	[0 1 3] 1.04(3)	[1 1 1] 0.57(2)	[1 1 1] 0.54(2)

Table S2. Refined atomic coordinates and their isotropic thermal parameters in various structural models

**$Cs_2NaInCl_6$**						
Atom	Wyckoff positions	*x/a*	*y/b*	*z/c*	Occupation	$B_{iso.}$, Å^2
Cs	8*c*	1/4	1/4	1/4	1	4.4(2)
Na	4*b*	1/2	1/2	1/2	1	2.9(11)
In	4*a*	0	0	0	1	3.4(3)
Cl	24*e*	0.2303(11)	0	0	1	3.6(3)
**$Cs_2Na_{0.97}In_{1.03}Cl_6$;** $Cs_2Na_{0.97}In^{+}_{0.03}In^{3+}Cl_6$						
Atom*	Wyckoff positions	*x/a*	*y/b*	*z/c*	Occupation**	$B_{iso.}$, Å^2
Cs	8*c*	1/4	1/4	1/4	1	4.6(2)
M1	4*b*	1/2	1/2	1/2	0.97 Na^+ + 0.03 In^+	3.9(11)
In	4*a*	0	0	0	1 In^{3+}	3.7(3)
Cl	24*e*	0.2303(11)	0	0	1	3.9(3)
**$Cs_2NaIn_{0.9}Sb_{0.1}Cl_6$**						
Atom*	Wyckoff positions	*x/a*	*y/b*	*z/c*	Occupation	$B_{iso.}$, Å^2
Cs	8*c*	1/4	1/4	1/4	1	3.58(11)
Na	4*b*	1/2	1/2	1/2	1	1.2(8)
M2	4*a*	0	0	0	0.9 In^{3+} + 0.1 Sb^{3+}	3.2(2)
Cl	24*e*	0.2370(10)	0	0	1	3.4(2)
**$Cs_2Na_{0.97}In_{0.93}Sb_{0.05}\square_{0.05}Cl_6$;** $Cs_2Na_{0.97}(In^{+})_{0.03}(In^{3+})_{0.9}(Sb^{3+/5+})_{0.05}\square_{0.05}Cl_6$						
Atom*	Wyckoff positions	*x/a*	*y/b*	*z/c*	Occupation**	$B_{iso.}$, Å^2
Cs	8*c*	1/4	1/4	1/4	1	5.3(2)
M3	4*b*	1/2	1/2	1/2	0.97 Na^+ + 0.03 In^+	1.8(8)
M4	4*a*	0	0	0	0.90 In^{3+} + 0.05 $Sb^{3+/5+}$ + 0.05 □	3.3(3)
Cl	24*e*	0.2362(9)	0	0	1	4.3(3)

*M1, M2, M3, M4 – mixture of the relevant cations; **In^+ and In^{3+} are distributed in different Wyckoff positions, while $Sb^{3+/5+}$ are in the same position.

Table S3. Interatomic distances and coordination numbers (C.N.) of atoms in the calculated structural models

Atoms	**$Cs_2NaIn^{3+}Cl_6$**	**$Cs_2Na_{0.97}In_{1.03}Cl_6$** $Cs_2Na_{0.97}(In^{+})_{0.03}In^{3+}Cl_6$	**$Cs_2NaIn^{3+}{}_{0.9}Sb^{3+}{}_{0.1}Cl_6$**	**$Cs_2Na_{0.97}In_{0.93}Sb_{0.05}\square_{0.05}Cl_6$** $Cs_2Na_{0.97}(In^{+})_{0.03}(In^{3+})_{0.9}(Sb^{3+/5+})_{0.05}\square_{0.05}Cl_6$	C.N.
	Interatomic distances, Å				
Cs–Cl	3.7307(7)	3.7308(7)	3.7289(4)	3.7292(4)	12
Na–Cl	2.841(12)	2.841(12)	2.772(10)	2.781(10)	6
In–Cl	2.427(12)	2.427(12)	2.498(10)	2.489(10)	6

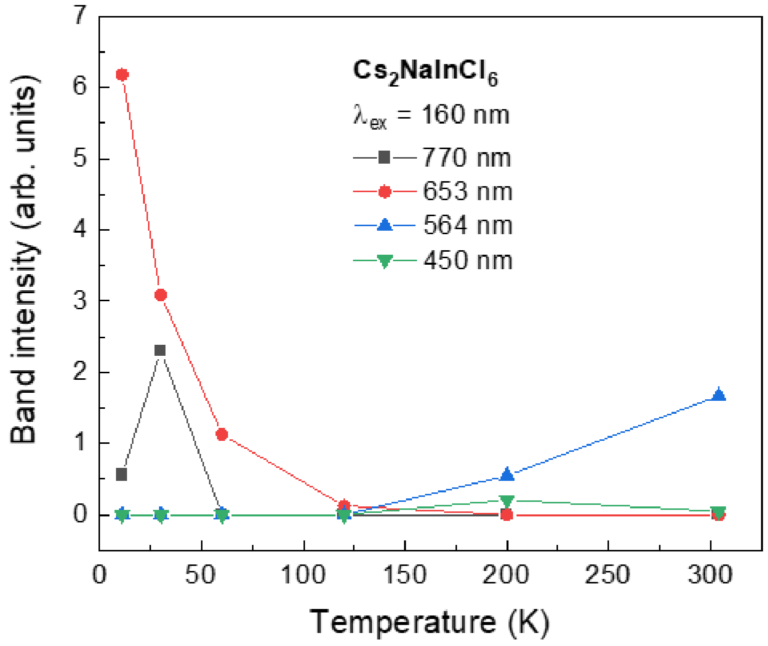

Fig. S3 Dependence of the CNIC PL band intensities on the temperature.

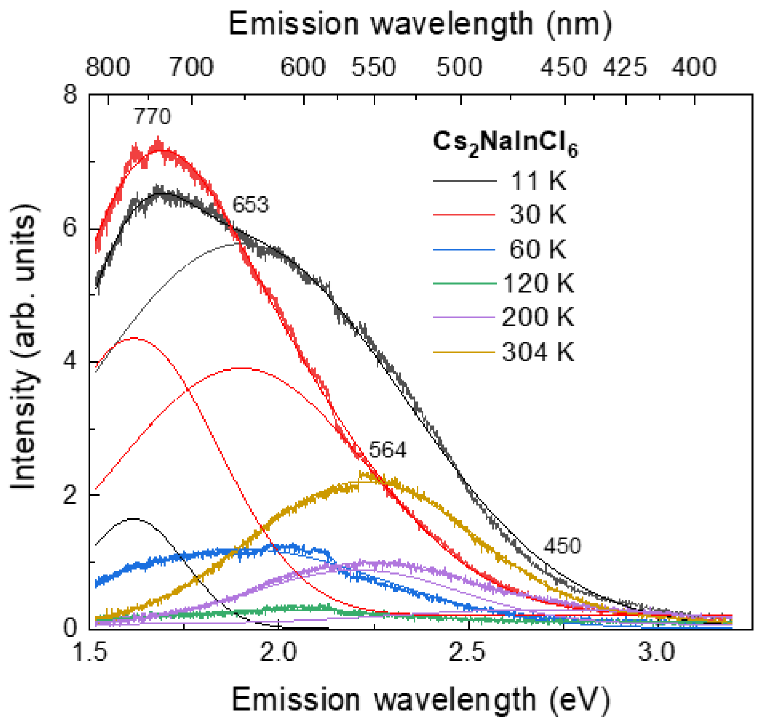

Fig. S4 The CNIC PL emission bands obtained at different temperatures.

Table S4. Lattice parameters and the total energy of the atom systems based on the CNIC crystal structure

Material	a, Å	Total energy, eV	Total energy/atom, eV
CNIC	10.3744	5787	144.68
CNIC + Er^{3+}	10.7100	4608	115.20
CNIC + Sb^{3+}	10.7560	4615	115.38
CNIC + V_{Cl}^{-}	10.7340	5796	148.62
CNIC + V_{Na}^{+}	10.6375	5490	140.77
CNIC + V_{In}^{3+}	10.6392	4634	118.82
$Cs_2Na_{1-x}In_{1+x}Cl_6$, $x = 0.06$	10.1695	5755	143.88
$Cs_2Na_{1-x}In_{1+x}Cl_6$, $x = 0.20$	10.2779	5600	140.00
$Cs_2Na_{1-x}In_{1+x}Cl_6$, $x = 0.50$	10.3328	4482	112.05
$Cs_2Na_{0.97}(In^{+}In^{3+})_{0.93}(Sb^{3+/5+})_{0.05}\square_{0.05}Cl_6$	10.6789	20.55	0.51

References:

(1) Akselrud L., Grin Yu., WinCSD: software package for crystallographic calculations (Version 4), *J. Appl. Crystallogr.* 2014, 47, 803.

(2) Kresse G., Joubert D. From Ultrasoft Pseudopotentials to the Projector Augmented-Wave Method. *Phys. Rev. B: Condens. Mater. Phys.*, 1999, 59, 1758-1775.

(3) Perdew J. P., Burke K., Ernzerhof M. Generalized Gradient Approximation Made Simple. *Phys. Rev. Lett.*, 1996, 77, 3865-3868.

(4) Monkhorst H. J., Pack J. D., Special points for Brillouin-zone integrations. *Phys. Rev. B.,*1976, 13, 5188–5192.
